# Zasp52 strengthens whole embryo tissue integrity through supracellular actomyosin networks

**DOI:** 10.1242/dev.201238

**Published:** 2023-04-03

**Authors:** Dina J. Ashour, Clinton H. Durney, Vicente J. Planelles-Herrero, Tim J. Stevens, James J. Feng, Katja Röper

**Affiliations:** ^1^ MRC Laboratory of Molecular Biology, Francis Crick Avenue, Cambridge Biomedical Campus, Cambridge CB2 0QH, UK; ^2^ Department of Mathematics, University of British Columbia, Vancouver, V6T 1Z2 Canada; ^3^ Department of Chemical and Biological Engineering, University of British Columbia, Vancouver, V6T 1Z3 Canada

**Keywords:** Actomyosin, Supracellular cable, Junctions, Cytoskeleton, Tension, Epithelium, Morphogenesis

## Abstract

During morphogenesis, large-scale changes of tissue primordia are coordinated across an embryo. In *Drosophila*, several tissue primordia and embryonic regions are bordered or encircled by supracellular actomyosin cables, junctional actomyosin enrichments networked between many neighbouring cells. We show that the single *Drosophila* Alp/Enigma-family protein Zasp52, which is most prominently found in Z-discs of muscles, is a component of many supracellular actomyosin structures during embryogenesis, including the ventral midline and the boundary of the salivary gland placode. We reveal that Zasp52 contains within its central coiled-coil region a type of actin-binding motif usually found in CapZbeta proteins, and this domain displays actin-binding activity. Using endogenously-tagged lines, we identify that Zasp52 interacts with junctional components, including APC2, Polychaetoid and Sidekick, and actomyosin regulators. Analysis of *zasp52* mutant embryos reveals that the severity of the embryonic defects observed scales inversely with the amount of functional protein left. Large tissue deformations occur where actomyosin cables are found during embryogenesis, and *in vivo* and *in silico* analyses suggest a model whereby supracellular Zasp52-containing cables aid to insulate morphogenetic changes from one another.

## Teaser

**Highlighted Article:** Zasp52 is a key component of supracellular actomyosin cables during epithelial morphogenesis that serve to insulate morphogenetic changes from each other.

## INTRODUCTION

Morphogenesis, the generation of shape in development, is the basis of organ formation and major topological changes in development. Within individual cells, changes in shape are driven by the cytoskeleton. Over the last decade, enormous progress has been made in deciphering which type of cell shape changes drive changes at the tissue scale (Blanchard et al., 2009; Guirao et al., 2015; Herrera-Perez and Kasza, 2018). Cell shape changes in most cases depend on the actomyosin cytoskeleton underlying the plasma membrane, the actomyosin cortex. This cortex is linked to cell surface receptors such as cadherins or integrins, thereby allowing modulation of the actomyosin cortex during cell-shape changes through cell-cell and cell-matrix adhesion (Chugh and Paluch, 2018; Mege and Ishiyama, 2017).

Crucially, cells do not change shape in isolation, rather a close coordination between all cells in a tissue is essential for productive changes at the tissue scale. Such coordination is based on different molecular mechanisms. Many morphogenetic events in embryos occur in epithelial tissues, where cell-cell adhesion between neighbouring cells allows for physical coupling of behaviours. The degree of turnover and stability of adhesion sites between neighbouring cells determines the extent of physical coupling between the neighbours, thereby, for example, controlling the number of neighbour exchanges (Song et al., 2013). Mechanical coupling through cell-cell adhesion connects the actomyosin cortices of neighbouring cells, which can also tie into cytoskeletal structures such as actin stress fibres or the microtubule cytoskeleton. Within epithelial cells, actomyosin is enriched near apical adherens junctions in what has been historically described as the ‘actin belt’ or ‘adhesion belt’ (Furukawa et al., 2017). Interestingly, this cell-cell-adhesion-associated actomyosin can be coordinated between neighbouring cells into seemingly ‘supracellular’ assemblies. These were first observed during wound healing in embryonic epithelia (Martin and Lewis, 1992), termed actomyosin ‘cables’ or ‘purse strings’, and subsequently found during morphogenetic processes across the evolutionary tree (Rodriguez-Diaz et al., 2008). Actomyosin cables have been intensively studied in model processes such as dorsal closure and salivary gland invagination in the *Drosophila* embryo (Jacinto et al., 2002; Röper, 2012; Sidor et al., 2020), neurulation in both *Ciona* (Hashimoto and Munro, 2019) and mouse (Galea et al., 2017), and wound healing (Wood et al., 2002). Actomyosin cables can show different properties and provide different functionalities: they can be stable, or dynamic and short lived, be involved in active morphogenetic changes or stable delineation of differently fated populations of cells (Röper, 2013). In a few instances, the upstream molecular cascade leading to the assembly of the cable has been elucidated (Hashimoto and Munro, 2019; Röper, 2012; Sidor et al., 2020).

We have previously analysed the morphogenesis of the tubes of the salivary glands from a flat epithelial primordium in the *Drosophila* embryo, termed the salivary gland placodes (Fig. S1), as well as the mechanism that leads to the assembly of supracellular actomyosin cables surrounding each of these two bilateral placodes (Röper, 2012; Sanchez-Corrales et al., 2018, 2021; Sidor et al., 2020). The salivary gland placodes are specified on the ventral side of the embryo half-way through embryogenesis at late stage 10, and cells start to invaginate through a focal point termed the invagination pit in the dorsal-posterior corner, forming a narrow lumen tube on the inside while cells continuously invaginate (Girdler and Röper, 2014; Sidor and Röper, 2016). Cells at the invagination point undergo apical constriction driven by apical-medial actomyosin prior to internalisation, while cells further away from the invagination pit undergo directional cell intercalations driven by polarised junctional actomyosin, to continuously feed cells towards the invagination point (Fig. S1A-I′; Sanchez-Corrales et al., 2018, 2021). Furthermore, the assembly of the supracellular actomyosin cable at the boundary of each placode with the surrounding epidermis is dependent on the anisotropic localisation of the transmembrane protein Crumbs and its downstream effectors Pak1 and aPKC (Röper, 2012; Sidor et al., 2020). The cable is under increased tension compared with nearby junctions that are not part of the cable, as shown by laser-ablation of junctions and measurements of initial recoil of junction vertices (Röper, 2012). The function of the circumferential cable surrounding the placode is not clear, but it could help the cell internalisation by exerting an inward-directed boundary force, or it could help to insulate cell behaviours within the primordium from the surrounding epidermis.

**Fig. 1.**
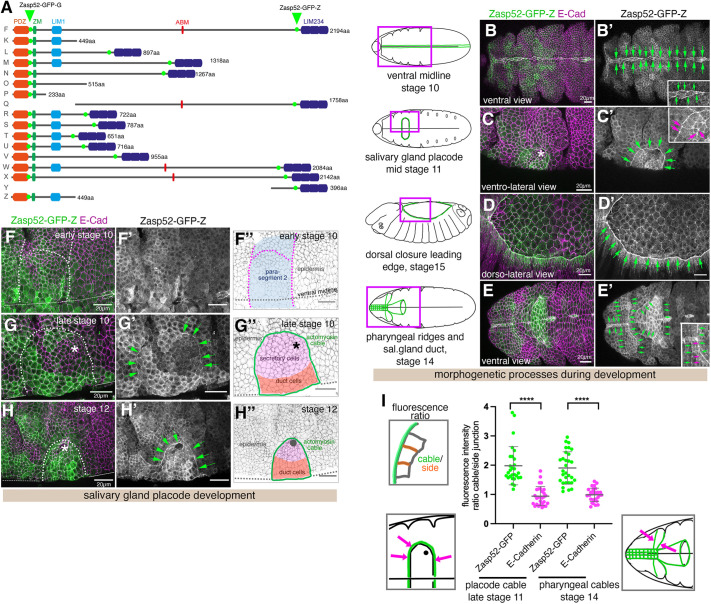
**Localisation of Zasp52 to actomyosin cables in the *Drosophila* embryo.** (A) Predicted protein isoforms of Zasp52, length in amino acids (aa) is given. The full-length protein contains an N-terminal PDZ domain, a Zasp-motif (ZM), a LIM domain (LIM1), a long unstructured region of varying length containing the newly identified actin-binding motif (ABM), followed by three C-terminal LIM domains (LIM 2-4). The insertion sites of GFP-protein traps Zasp52-GFP [G00189] (G) and Zasp52-GFP [ZCL423] (Z) are indicated in green. (B-E′) Localisation of Zasp52-GFP-Z to supracellular actomyosin cables: the cables lining the ventral midline (B,B′), the cable surrounding the salivary gland placode (C,C′), the leading edge epidermal cable during dorsal closure (D,D′), other ventral actomyosin cables during head involution surrounding the pharyngeal ridges and cells forming the salivary gland duct (E,E′). Magenta boxes in schematics indicate positions of images taken, green lines indicate actomyosin cables shown in the images. Green arrows in B′-E′ indicate the position of the actomyosin cables containing Zasp52. Insets show higher magnifications of sections of the images. Magenta arrows in C′,E′ (insets) point to accumulation of Zasp52 in tricellular junctions. (F-H″) Localisation of Zasp52-GFP-Z in the salivary gland placode showing a patchy pattern during specification of the tissue in parasegment 2 (F-F″), starting accumulation at the boundary cable from late stage 10 (G-G″) and strong accumulation at later stages (H-H″). F″-H″ show schematics of stages, cell types (secretory cells, pink; duct cells, orange), position of the forming invagination pit (asterisk in G″) and the actomyosin cable at the boundary (green). Green arrows in G′ and H′ point to Zasp52 accumulation in the cable. Zasp52-GFP-Z is in green, E-Cadherin to label cell outlines is in magenta. Dotted lines in F-H show the boundary of the salivary gland placode. Asterisk in G and H shows the position of the invagination point. (I) Quantification of Zasp52-GFP enrichment in cable junctions compared with side junctions of cells at boundaries, see schematic, where actomyosin cables assemble. Quantified are the Zasp52-GFP enrichment compared with E-Cadherin in the same junctions in the salivary gland placode cable at late stage 11 (28 junctions from three embryos) and the anterior pharyngeal cables at stage 14 (30 junctions from three embryos) as indicated in the accompanying schematics, with magenta arrows pointing to the respective cables. Data are mean±s.d. ****P<0.0001 (paired two-tailed Student's *t*-test). Scale bars: 20 µm. See also Figs S1 and S2.

Zasp52 is a member of the Alp/Enigma family of proteins, containing an N-terminal PDZ domain and four LIM domains (Fig. 1A). *Drosophila* Zasp52 was originally identified as a component of striated muscles, localised to the Z-line, where it interacts with its binding partner α-actinin and is important to link actin-barbed ends into the Z-disc (Jani and Schöck, 2007). More recently, Zasp52 was also found to localise to the actomyosin cable that assembles at the leading front of epidermal cells during dorsal closure in the fly embryos (Stronach, 2014). These cells move towards the dorsal side of the embryo to cover the non-embryonic tissue of the amnioserosa. Zasp52 was shown to assist in providing a straight leading-edge front, thereby assisting the correct matching of segments between left and right sides of the embryo (Ducuing and Vincent, 2016).

Here, we show that Zasp52 is in fact a component of many, though not all, embryonic actomyosin cables or supracellular actomyosin assemblies. Zygotic loss of Zasp52 leads to a reduction of junctional F-actin in the salivary gland placodal cable. We identify a novel F-actin binding motif (ABM) in the coiled-coil domain of Zasp52 that is related to the motif usually found the in the actin-capping protein CapZbeta. In addition, we identify several ubiquitous cell-cell-adhesion-associated proteins as interaction partners of Zasp52, in particular APC2, Polychaetoid (Pyd; ZO-1) and Sidekick (Ahmed et al., 2002; Choi et al., 2011; Finegan et al., 2019; Letizia et al., 2019). Embryos lacking all maternal and zygotic Zasp52 function show major defects in morphogenetic events in tissues associated with the presence of supracellular actomyosin cables such as the salivary glands, the ventral midline, the head and the leading edge/amnioserosa interface. We propose that the supracellular actomyosin cables containing Zasp52 serve as mechanical insulators, preventing morphogenetic changes from ‘spilling over’ into neighbouring regions or otherwise interfering with nearby events, a model which is further supported by *in silico* investigations.

## RESULTS

### Zasp52 is a component of embryonic supracellular actomyosin cables

In order to analyse Zasp52 protein localisation in the *Drosophila* embryo we made use of two protein-trap insertion lines in the *zasp52* locus (Fig. 1A). Zasp52-GFP (using either the Zasp52[ZCL423] or Zasp52[G00189] lines) within the embryonic epidermis of *Drosophila* was particularly strongly localised to junctions that were part of supracellular actomyosin assemblies or cables (Fig. S1F-H). Both GFP-exon trap lines label most isoforms of Zasp52, including the longest possible one (Fig. 1A, protein isoform F) and both lines in the embryo show indistinguishable patterns (Fig. S2C-H′). Zasp52-GFP was enriched in junctions of the ventral midline (Fig. 1B,B′), the cable surrounding the salivary gland placode (Fig. 1C,C′), the leading edge of epidermal cells during dorsal closure (Fig. 1D,D′) as well as several large-scale cables found on the ventral anterior side of the embryo at the start of head involution (Fig. 1E,E′; Röper, 2013). These ventral anterior cables in particular appeared to be linked into a large-scale network that spans the whole anterior epidermis at this stage (arrows in Fig. 1E′). We could not detect Zasp52-GFP in cables found at parasegmental boundaries in the early embryo before stage 11 or in the more dynamic short cables found in tracheal placodes during tracheal morphogenesis (Fig. S2). In addition to supracellular cables, Zasp52-GFP localised at lower levels to some bicellular junctions and was always slightly more enriched at tricellular junctions (highlighted in Fig. 1C′ and E′, magenta arrows in insets).

We analysed in more detail the localisation and levels of Zasp52-GFP in the forming salivary gland placode. Zasp52-GFP was only present at low levels in the embryonic epidermis at early stage 10 before placode specification (Fig. 1F-F″), but showed a patchy expression in the salivary gland placode at late stage 10, with accumulation at circumferential cable commencing at this stage (Fig. 1G-G″). During stages 11 and 12, Zasp52-GFP quickly accumulated in the forming cable at the placode boundary (Fig. 1H-H″). In fact, by late stage 11 Zasp52-GFP was strongly enriched in the placodal cable compared with E-Cadherin (also known as Shotgun, Shg) (Fig. 1I), showing a clear anisotropy in the boundary cells that was as prominent as the anisotropy of Rok or myosin (Röper, 2012; Sidor et al., 2020). The enrichment in the ventral anterior cables at stage 14 was of similar magnitude (Fig. 1I). Zasp52 therefore appears to be an enriched component of many, though not all, supracellular actomyosin cables in the *Drosophila* embryo.

### Zasp52 contains an actin-binding motif related to the CapZbeta-actin binding motif

**Fig. 2.**
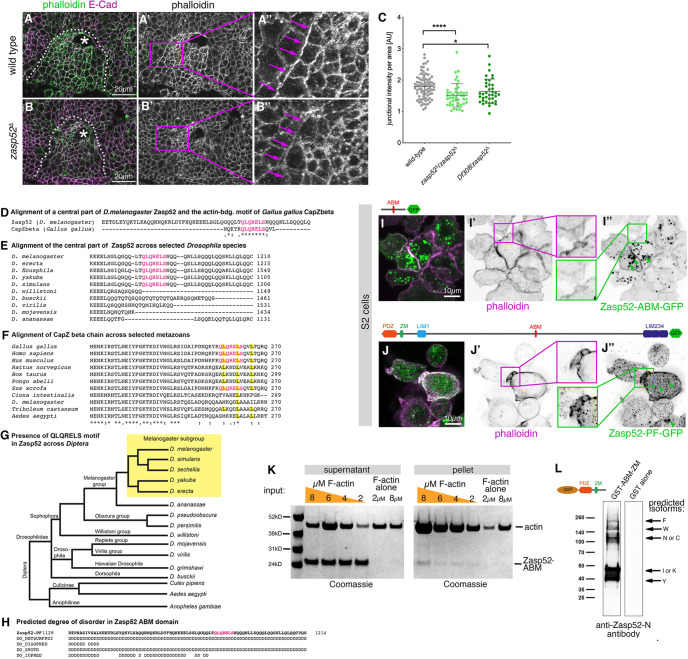
**Zasp52 contains a novel F-actin binding site related to CapZbeta and affects supracellular F-actin localisation.** (A-B″) F-actin accumulates at the boundary of the salivary gland placode where a supracellular actomyosin cable forms (A-A″). In *zasp52*^Δ^ zygotic mutants less F-actin accumulates (B-B″). E-Cadherin, magenta; Phalloidin (F-actin), green. Asterisks mark the invagination point. The dotted lines indicate the boundary of the placode. (C) Quantification of Phalloidin intensity at the boundary of the placode in the wild-type compared with *zasp52*^Δ^ homozygous mutants or *zasp52*^Δ^*/Df308* transheterozygous mutants (*Df308* uncovers the *zasp52* locus). Data are mean±s.d. *P<0.05, ****P<0.0001 (two-tailed Mann–Whitney test). (D) Pairwise alignment of CapZbeta (*Gallus gallus*) and Zasp52 (*Drosophila melanogaster*), conserved motif highlighted in pink. This motif in Zasp52 is near identical to the actin-capping motif in CapZbeta. (E) Amino acid sequence alignment of Zasp52-PF across members of the *Drosophila* genus. Conserved, putative ABM highlighted in pink. (F) Amino acid sequence alignment of CapZbeta across metazoan species with conserved leucines of CapZbeta highlighted in yellow (Hug et al., 1992; Kim et al., 2010) and the complete QLQRELS motif (pink letters). (G) Phylogenetic tree of the *Drosophila* genus with *D. melanogaster* subgroup (yellow) in which species show the QLQRELS motif in Zasp52. In D and F, the asterisk indicates positions which have a single, fully conserved residue; the colon shows conservation between groups of strongly similar properties; and the period shows conservation between groups of weakly similar properties. (H) Secondary structure predictions about local disorder in the stretch of amino acids surrounding the ABM in Zasp52-PF, generated with Quick2D toolkit (NeetSurfP2 (Klausen et al., 2019), DISOPRED3 (Jones and Cozzetto, 2015) and SPOT-Disorder (Hanson et al., 2017), suggesting this region to be disordered (D=disorder). (I-I″) Ectopic expression of the Zasp52-ABM-GFP in S2 cells, where it localises to the cell cortex (I″, green) and colocalises with F-actin (I′; Phalloidin, magenta). (J-J″) Ectopic expression of the long Zasp52-PF-GFP isoform in S2 cells, where it localises to the cell cortex (J″, green) and colocalises with F-actin (J′; Phalloidin, magenta). Both constructs also show small non-specific aggregates in the centre of the expressing cells (green arrowheads in I″ and J″). (K) Ultracentrifugation-based co-sedimentation assay of F-actin and Zasp52-ABM: 0.8 µM Zasp52-ABM was incubated with increasing concentrations of pre-polymerised F-actin (2 µM, 4 µM, 6 µM, 8 µM). Blots of supernatant and pellet were analysed by staining with Instant Blue (Coomassie) dye. The 2 µM and 8 µM Zasp52-ABM were centrifuged without F-actin as control. (L) Pull-down of bound Zasp52 isoforms using GST-Zasp52-PDZ-ZM as bait, labelled with anti-Zasp52-N antibody, indicating that this domain likely participates in dimerisation of Zasp52. Scale bars: 20 µm. See also Fig. S3.

The strong enrichment of Zasp52-GFP in supracellular actomyosin structures and especially the circumferential cable around the salivary gland placode prompted us to investigate whether Zasp52 played a role in the establishment or function of this cable. In wild-type embryos, not only myosin II (Fig. S1K-K″) but also F-actin (Fig. 2A-A″; Röper, 2012) accumulates strongly at the boundary of the placode. In embryos zygotically lacking Zasp52, using a deletion of *zasp52* called *zasp52*Δ (Jani and Schöck, 2007), this F-actin accumulation at the placode boundary was strongly reduced, as it was in transheterozygous embryos of the *zasp52*Δ allele combined with an allele of a deficiency spanning the *zasp52* locus (Fig. 2B-C).

Zasp52 has been suggested to interact with F-actin directly through its N-terminal region, containing the PDZ domain and Zasp motif (Liao et al., 2020). Using a purified Zasp52 fragment of this region (PDZ+ZM) we confirmed *in vitro* that this binding is direct and does not require additional factors (Fig. S3). Furthermore, in the Z-disk of muscles, Zasp52 binding to actin is mediated by its interactor α-actinin (Jani and Schöck, 2007). However, using a GFP-protein-trap insertion into the α-actinin locus we found that α-actinin-GFP in embryos did not localise to junctions of epidermal cells and was not enriched in the cable surrounding the salivary gland placode (data not shown). As actin-binding through the PDZ+ZM part of Zasp52 appeared weak, we analysed the amino acid sequence of the longest isoform of Zasp52 (isoform PF) for further possible conserved actin-binding motifs that might have been overlooked. We identified a motif in the central unstructured region of Zasp52 that closely resembles the highly conserved actin-capping-motif usually found in the actin-capping protein CapZbeta (Fig. 2D-G; Hug et al., 1992; Kim et al., 2010). In CapZbeta, the conserved residues are part of a flexible C-terminal domain shown to be able to flip in and out of the hydrophobic furrow of the F-actin analogue Arp1-A. This interaction is integral to the capping of the barbed end of F-actin (Narita et al., 2006; Urnavicius et al., 2015; Yamashita et al., 2003). Although the motif in Zasp52 is not located at the C-terminus, it is in an area of higher disorder implying increased flexibility (Fig. 2H). This motif in Zasp52 is not only found in *D. melanogaster*, but conserved in a subset of *Drosophilidae* (Fig. 2G).

We expressed the Zasp52-ABM (see location in Fig. 1A) fused to GFP in S2 cells, where it colocalised with cortical F-actin structures labelled using Phalloidin (Fig. 2I-I″), though it also formed non-specific aggregates, likely due to the overexpression. Furthermore, the Zasp52-PF isoform, containing the ABM in its central part, also colocalised with F-actin labelled by Phalloidin in S2 cells (Fig. 2J-J″), but also showed non-specific aggregation. We then purified recombinant Zasp52-ABM and performed *in vitro* actin-binding assays. Zasp52-ABM pelleted with F-actin in an F-actin-dose dependent manner, as would be expected from a capping protein (Fig. 2K).

In addition, we uncovered that a GST-tagged N-terminal fragment of Zasp52 (PDZ+ZM) can co-precipitate several untagged endogenous Zasp52 isoforms from embryonic lysate (Fig. 2L), supporting that this domain is involved in Zasp52 dimerisation or multimerisation, which has been suggested to be dependent on the interaction of the Zasp motif with the LIM1 domain (Gonzalez-Morales et al., 2019).

Thus, Zasp52 is important for F-actin accumulation in the cable surrounding the salivary gland placode, possibly via the N-terminal as well as the newly-identified ABM in its central region. The ability to dimerise or multimerise suggests that Zasp52 might not only bind and cap actin filaments with the two respective ABMs, but that it could also assist in actin cross-linking, actin bundling or other higher order organisation of F-actin filaments, thereby possibly affecting actomyosin contractility at the site of supracellular actomyosin structures.

### Zasp52 associates with junctional proteins

**Fig. 3.**
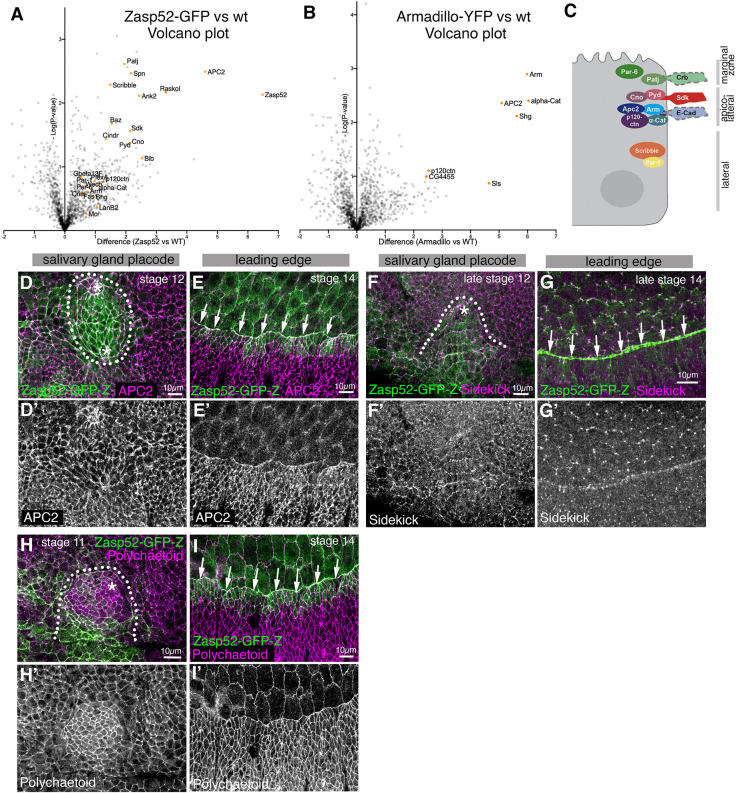
**Zasp52-GFP interacts with many junctional proteins.** (A,B) Volcano plots comparing spectral intensity values generated from anti-GFP co-immunoprecipitations from embryo lysates of Zasp52-GFP, Armadillo-YFP and wild-type embryos. Three experiments each for Zasp52-GFP and wild-type embryos were performed and two for Armadillo-YFP embryos. A shows enriched interactors for Zasp52-GFP compared with wild type (highlighting hits related to cell adhesion and regulation of junctional cytoskeleton), and B shows enriched interactors for Armadillo-YFP compared with wild type. The *x*-axis is the difference score for each protein as calculated in MaxQuant, and *y*-axis is the probability score calculated between Zasp52-GFP or Armadillo-YFP and wild type. The significance threshold was set to 0.75. (C) Schematic of protein complexes and interactions in marginal zone, apico-lateral and lateral junctions in *Drosophila* epithelial cells. (D-I′) Localisation of three possible Zasp52-interaction partners within the embryonic epidermis. Localisation around the salivary gland placode and in the leading edge epidermal cells and amnioserosa during dorsal closure is shown, two processes with prominent supracellular actomyosin cables containing Zasp52. (D-E′) APC2 localises to apical junctions, both bicellular and tricellular, but is more enriched at tricellular junctions. (F-G′) Sidekick is particularly enriched in tricellular junctions. (H-I′) Polychaetoid is localised homogeneously to apical junctions throughout the epidermis. APC2, Sidekick and Polychaetoid are not enriched in actomyosin cables. Zasp52-GFP, green; APC2, Sidekick and Polychaetoid, magenta. Dotted lines indicate the boundary of the salivary gland placode; asterisks mark the position of the invagination point; arrows indicate the position of the cable during dorsal closure. Scale bars: 10 µm. See also Fig. S4 and Tables S1 and S2.

In order to identify whether and how Zasp52 was recruited specifically to supracellular actomyosin structures and uncover what function it fulfils there, we set out to identify potential further interaction partners in addition to F-actin. We generated lysate from embryos before formation of muscles (pre stage 14), either from embryos expressing endogenously-tagged Zasp52, *Zasp52[ZCL423]*, or from wild-type embryos, or from embryos expressing an endogenously-tagged Armadillo/β-Catenin, *armadillo-YFP*, and performed anti-GFP co-immunoprecipitations (note that as soluble cold lysates were used, no F-actin or polymerised microtubules are present in the input material). Anti-GFP immunoprecipitation of Zasp52-GFP lysates compared with wild-type lysates showed a specific enrichment of many potential interactors with links to both actomyosin and cell-cell adhesion (Fig. 3A; i.e. APC2, Sidekick, Polychaetoid, Patj, Raskol, Canoe, Cindr, Bazooka, p120-Catenin, α-Catenin, G-beta13F, Scribble), whereas the anti-GFP immunoprecipitation from Armadillo-YFP lysates compared with wild-type lysates identified the components of adherens junctions known to interact with Armadillo as enriched (Fig. 3B; i.e. Armadillo, α-Catenin, Shotgun, APC2, p120-Catenin). The identification of partly distinct sets of interactors for Zasp52-GFP and Armadillo-YFP indicated that Zasp52 is not just a direct interactor of adherens junctions (Fig. 3A-C; for a full list of interactors see Tables S1 and S2).

We analysed the localisation of potential interaction partners in comparison with Zasp52-GFP, in particular the localisation of the junctional components APC2, Polychaetoid and Sidekick (Ahmed et al., 2002; Choi et al., 2011; Finegan et al., 2019; Letizia et al., 2019). APC2, one of the two APC tumour suppressor proteins in *Drosophila* with functions in Wnt signalling and cell adhesion (Ahmed et al., 2002; Hamada and Bienz, 2002), was localised to junctions in the embryonic epidermis and was particularly enriched at tricellular junctions, but was not specifically enriched in supracellular actomyosin structures (Fig. 3D-E′). However, it colocalised with Zasp52-GFP in the junctions where Zasp52 was enriched. Similarly, Sidekick, a protein especially enriched in tricellular junctions in the embryonic epidermis and important for epithelial integrity during morphogenesis (Finegan et al., 2019; Letizia et al., 2019), also colocalised with Zasp52-GFP in these junctions (Fig. 3F-G′). Lastly, Polychaetoid, a protein with a variety of functions in apical junctions including linkage to the actin cytoskeleton (Choi et al., 2011), localised to apical junctions in the embryonic epidermis and was enriched in placodal junctions, again colocalising there with Zasp52-GFP (Fig. 3H-I′).

Thus, Zasp52-GFP appeared to associate with many junctional proteins at bi- and tricellular junctions. When analysing its localisation along the apical to basal extent of the epithelial junctions, Zasp52 in actomyosin cables colocalised with E-Cadherin at the level of adherens junctions, but also partially overlapped with the more apically localised Crumbs protein within the apical marginal zone of the lateral sides (Fig. 3C; Fig. S4A-F).

The associated proteins analysed above do not usually show a specific enrichment in supracellular junctional actomyosin structures, suggesting that the localisation to and incorporation of Zasp52 into such structures is likely controlled by its pattern of expression and its general ability to bind F-actin and junctional components, rather than a unique membrane-associated binding partner in these supracellular assemblies. In agreement with this, overexpression of different Flag-tagged Zasp52 isoforms in stripes in the embryonic epidermis, using *enGal4*, led to junctional localisation of these isoforms (Fig. S4G-H′), confirming that Zasp52 binding partners appear to be present in epidermal junctions throughout the embryo.

### Partial and complete loss of Zasp52 affects epithelial morphogenesis

**Fig. 4.**
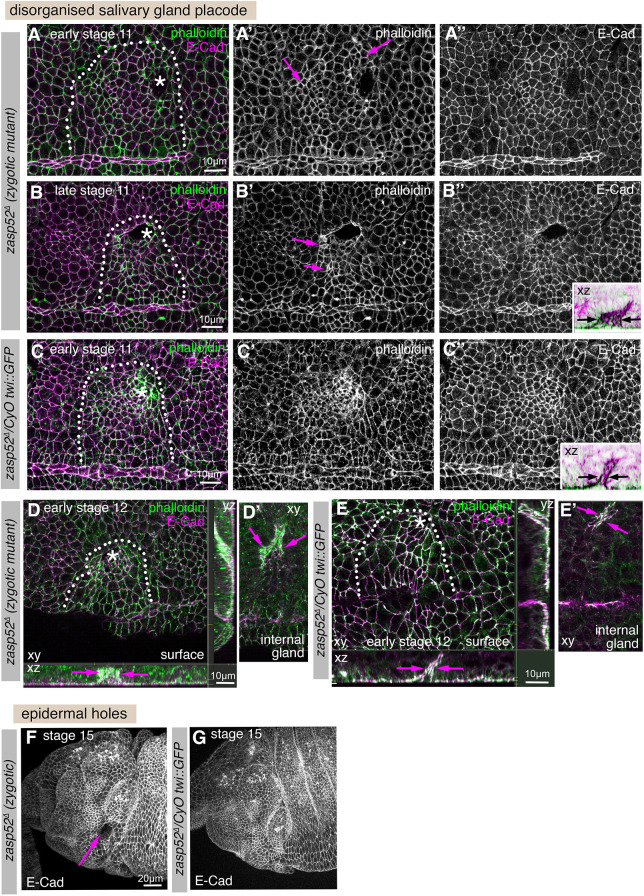
**Zygotic loss of Zasp52 function leads to mild embryonic defects.** (A-C″). Salivary gland placodes in *zasp52*^Δ^ (zygotic) mutants at stage 11 show slight disorganisation with the pattern of apical constriction (magenta arrows in A′,B′) not matching control placodes (C-C″), and invaginated glands show aberrant lumen shapes already at this stage (compare width of invagination in inset in B″ with control in inset in C″). E-Cadherin, magenta; Phalloidin, green. White dotted lines mark the boundary of the placode; asterisks indicate the position of the invagination pit. Insets in B″ and D″ are in inverse colour to better illustrate the lumen shape (black arrows indicate width of aberrant versus normal lumen diameter). (D-E′) At stage 12, invaginated lumens in *zasp52*^Δ^ mutants are often aberrantly shaped, as evident in xz and zy cross-sections (D compared with control in E) and internal xy sections below the epidermis (D′ compared with control in E′). Magenta arrows indicate width of aberrant versus normal lumen diameter. (F,G) At stage 15, when the salivary glands have completely invaginated, a third of *zasp52*^Δ^ mutant embryos show an epidermal hole where the salivary gland would have previously invaginated from the surface (magenta arrow, F). This is never seen in the wild-type (G). Labelling is E-Cadherin to mark apical cell outlines. Scale bars: 10 µm (A-E′); 20 µm (F,G).

In order to uncover the molecular role of Zasp52 in supracellularly coordinated junctions, we analysed the embryonic phenotypes, first in the zygotic mutant *zasp52*^Δ^, in more detail. *zasp52*^Δ^ embryos, in addition to the reduction in F-actin accumulation at the salivary gland placode boundary shown above (Fig. 2A-C), showed a slight disorganisation of the placode in that some groups of cells appeared to be over-constricted (Fig. 4A′,B′, magenta arrows) compared to control where there is a clear gradient of constriction from the invagination point (Fig. 4C-C″; Sánchez-Corrales and Röper, 2018). Invaginated glands often showed an aberrant lumen (Fig. 4B″ inset, compared with control in Fig. 4C″ inset, Fig. 4D-E′). Such a widened invagination pit is indicative of defects in coordination of cell behaviours such as apical constriction during invagination (Sanchez-Corrales et al., 2021). At mid to late stages of embryogenesis, *zasp52*^Δ^ embryos showed occasional holes within the epidermis where the salivary glands previously invaginated into the embryo (Fig. 4F compared with wild type in G, magenta arrow).

**Fig. 5.**
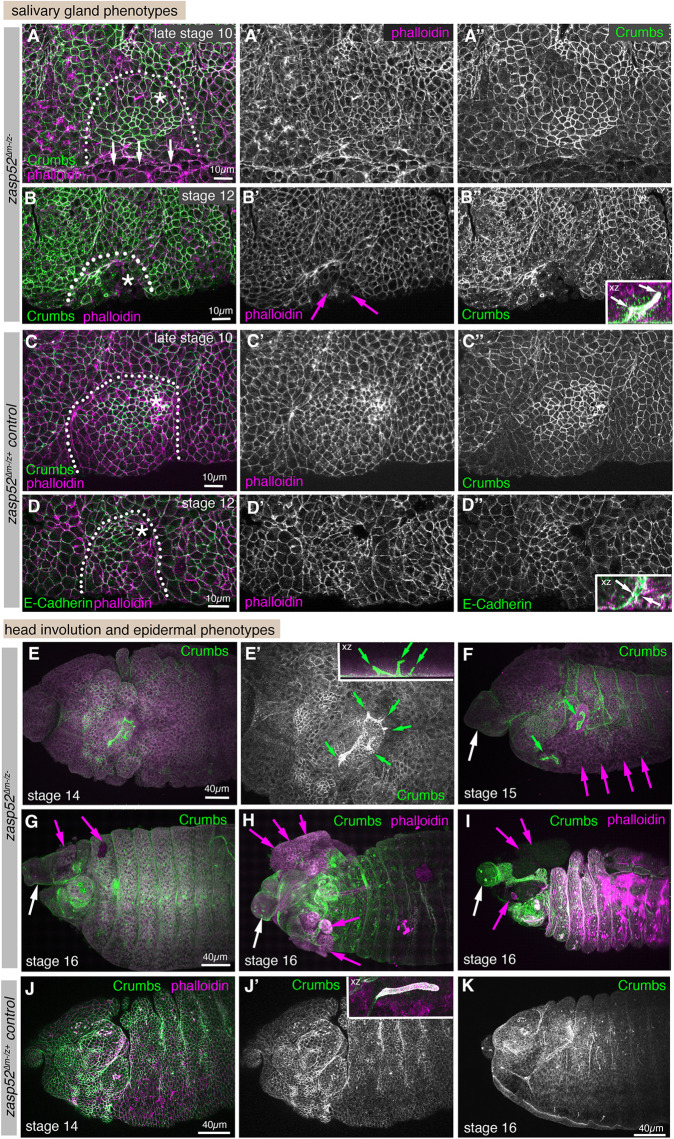
**Complete loss of Zasp52 function severely disrupts morphogenetic processes.** (A-D″) Salivary gland placodes in *zasp52*^Δ*m−/z−*^ (maternal and zygotic) mutant embryos show disorganised salivary gland placodes with a disrupted boundary (A-A″), including a disorganised ventral midline (white arrows in A) and also epidermal tears in close proximity to where the supracellular actomyosin cable should be localised or where remnants of it remain (B-B″, arrows point to tears). Wild-type placodes, by contrast, show the graded pattern of apical constriction expected at late stage 10 and a smooth boundary (C-D″). Invaginated salivary glands show highly aberrant lumens (B″, inset, and E′,F), compared with the smooth narrow lumen of the control (D″ inset). White arrows in inset in B″ point to branched lumen, and to the narrow lumen in the control in D″. Dotted lines mark the boundary of the placode, asterisks mark the position of the invagination point. (E-K) *zasp52*^Δ*m−/z−*^ mutants at later embryonic stages 14-16 show severe problems with head involution, with major anterior parts failing to internalise (white arrows), compared with the control (J,J′) and tears and holes appearing in the epidermis through which internal structures protrude (magenta arrows) that are also absent in the control (J,J′). Note the aberrant and branched lumens of the salivary glands (green arrows) compared with the narrow unbranched lumen in the control (inset in J′). Epidermal tears and holes in maternal zygotic mutants were paternally rescued (m^−^/z^−^: 13/35; m^−^/z^+^: 1/20). Deformations due to failed head involution or loss of midline structures were prevalent in maternal zygotic mutant embryos (24/35 m^−^/z^−^embryos) and only paternally rescued in half of these (7/20 m^−^/z^+^ embryos still showed the phenotype). Crumbs, green; Phalloidin, magenta. Scale bars: 10 µm (A-D″); 40 µm (E-K).

As *zasp52* mRNA is provided maternally (https://insitu.fruitfly.org/cgi-bin/ex/insitu.pl; Tomancak et al., 2002), in order to observe the most severe possible phenotypes, we generated embryos lacking both maternal and zygotic Zasp52 contribution, *zasp52*^Δ*m−/z−*^ embryos. These embryos completely lacking Zasp52 displayed numerous defects, including a strong loss of patterned cell shapes in the early salivary gland placode compared with control placodes (Fig. 5A-A″ versus C-C″) or even holes or tissue disruption where the placode is located (Fig. 5B-B″, arrows in B′, compared to D-D″), as well as aberrant lumens of glands that managed to invaginate (Fig. 5B″ inset versus D″ inset; E,E′ at stage 14 compared with control in J,J′). Later stage embryos often showed wider epidermal disruption, in particular in regions where supracellular actomyosin cables were present in wild-type embryos such as the head region and ventral midline, whereas the posterior epidermis and segmental pattern appeared to be unaffected. Defects were visible as the disruption or lack of ventral midline structures (already visible at stage 10 in Fig. 5A, arrows, and loss of the ventral midline at stage 15 in Fig. 5F). Furthermore, head involution, a process whereby the anterior-most structures of the embryo are internalised in major morphogenetic movements, appeared to partially fail, with structures that should have been internalised remaining on the outside along with significant epidermal tears visible in these regions (Fig. 5F-I, white arrows pointing to non-internalised structures, magenta arrows pointing to epidermal holes and tears, compare with control in K). These phenotypes are reminiscent of disruption seen in weak alleles of *shg* (E-Cadherin) or alleles of α-Catenin that cannot interact with E-Cadherin and thus disrupt the link between E-Cadherin and actin (Orsulic and Peifer, 1996; Tepass et al., 1996), in line with Zasp52 also playing a role in the linkage of actin to junctions.

Therefore, loss of Zasp52 appeared to impair morphogenesis of tissues that displayed prominent supracellular actomyosin cables during their morphogenesis and led to large-scale disorganisation of embryonic tissues. The observed phenotypes of major aberrant tissue deformations as well as epidermal ruptures suggest that these cables could have served to coordinate major movements or protect tissue integrity.

### Genetic interaction suggests cooperation between Zasp52 and APC2

We identified above that Zasp52 is able to physically associate with several junctional components. We therefore generated a double mutant line lacking both Zasp52 and APC2, *zasp52*^Δ^*; apc2^D40^*, to analyse how this affected embryonic development*.*

Loss of APC2 alone within the embryonic epidermis, in *apc2^D40^* and *apc2^N175K^* mutant embryos, led to phenotypes at the salivary gland placode boundary and at the leading edge-amnioserosa interface that suggested an imbalance of forces of contracting cells with their neighbours (Fig. S5). For example, cells within the salivary gland placode appeared to be over-constricted, whereas in the epidermis just outside the placode boundary, cells were overstretched compared with control (Fig. S5A-B″). This could suggest a weakened adherens junction-actomyosin link in these mutants.

**Fig. 6.**
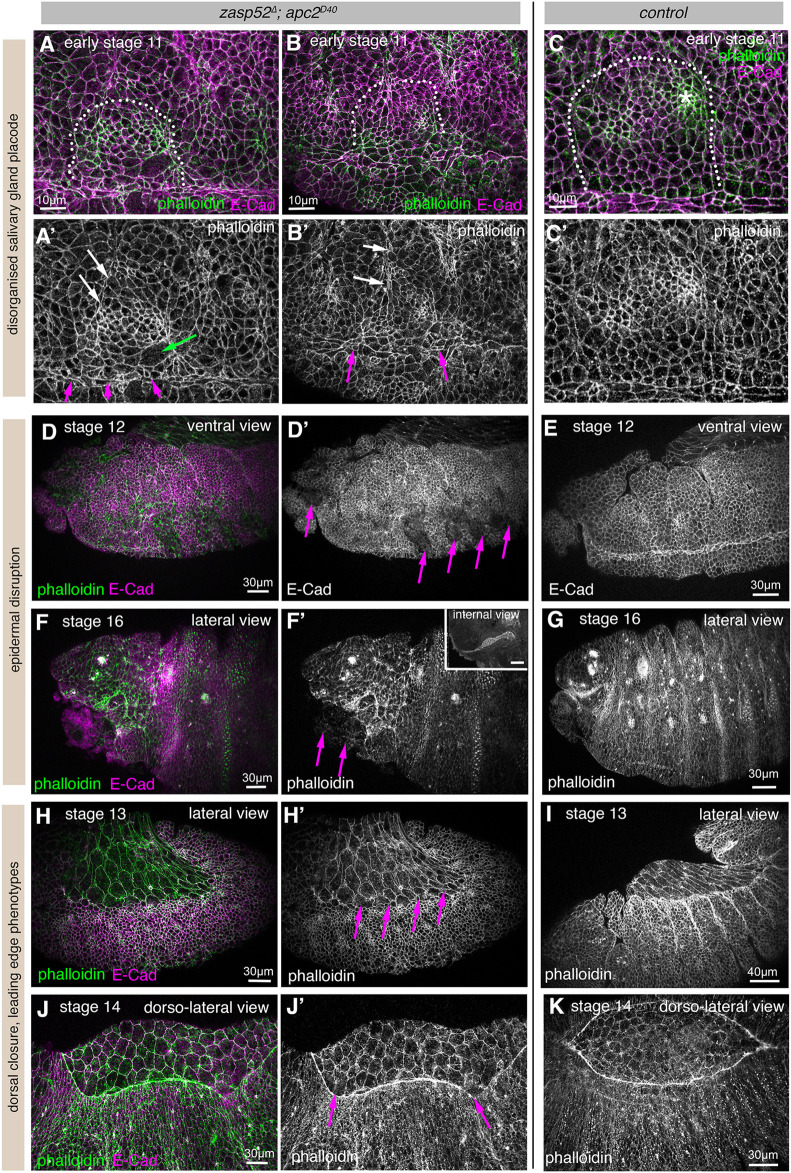
**Zasp52 and APC2 interact genetically to strengthen supracellular actomyosin assemblies.** (A-C′) Salivary gland placodes in *zasp52*^Δ^*; apc2^D40^* mutant embryos are disorganised (A-B′) with aberrant patterns of apical constriction (green arrow), a disorganised boundary and ventral midline (magenta arrows) and the maximillar-mandibular fold invading the placodal area (white arrows), in comparison with control placodes (C,C′). Dotted lines mark the position of the boundary of the placode. Asterisk indicates the position of the invagination pit. (D-G) In *zasp52*^Δ^*; apc2^D40^* mutants at stage 12 (mid-embryogenesis; D,D′) the epidermis near the ventral midline is often disrupted by tears (magenta arrows in D′), compared with the smooth intact epidermis in control embryos (E). At late stage 16, the anterior head region in *zasp52*^Δ^*; apc2^D40^* mutants shows tears and protruding internal tissues (F,F′; magenta arrows) compared with the intact epidermis in control embryos (G). The invaginated salivary gland of the embryo shown in F,F′ also has an aberrant lumen shape (inset in F′). (H-I) Before dorsal closure commencing, *zasp52*^Δ^*; apc2^D40^* mutants at early stages can fail to develop a clear boundary between epidermis and amnioserosa (magenta arrows in H′) compared with control embryos (I). (J-K) During dorsal closure *zasp52*^Δ^*; apc2^D40^* mutants (J,J′) show uneven accumulation of F-actin in the leading edge cable and deformation of the cable indicative of uneven tension (magenta arrows in J′) compared with the homogeneous accumulation in control embryos (K). E-Cadherin, magenta; Phalloidin, green. Scale bars: 10 µm (A-C′); 30 µm (D-H′,J-K); 40 µm (I). See also Fig. S5.

Embryos double-zygotic mutant for Zasp52 and APC2, *zasp52*^Δ^*; apc2^D40^*, similar to embryos lacking both maternal and zygotic Zasp52, displayed disrupted salivary gland placodal organisation (Fig. 6A-B′, compare with C,C′), with cells displaying aberrant apical areas (green arrow in Fig. 6A′), and showed a disorganised ventral midline (Fig. 6A′,B′, magenta arrows). Also, the dorsally-located fold between maximillar and mandibular segments protruded erroneously into the placodal area (Fig. 6A′,B′, white arrows). Furthermore, similar to the *zasp52*^Δ*m−/z−*^ mutants, tears and holes appeared mid-embryogenesis around the ventral midline (Fig. 6D,D′), and late in embryogenesis in the anterior part of embryos, again with head involution being impaired (Fig. 6F,F′), compared with control embryos of matching stages (Fig. 6E,G). In addition, the formation and closure of the amnioserosa and its surrounding actomyosin cable was affected (Fig. 6H-J′). At early stages during germband retraction, in some embryos, no clear boundary between the epidermis and amnioserosa developed (Fig. 6H′, magenta arrows, compare with I). In others, despite accumulation of F-actin at the leading edge front in many, though not all, cells the leading edge front was not taut, possibly due to an imbalance of tension (Fig. 6J,J′, magenta arrows; compare with K).

In contrast to the milder phenotypes observed in *zasp52*^Δ^ zygotic mutants, the embryos double-mutant for *zasp52*^Δ^ and *apc2^D40^* showed phenotypes more similar to those observed in *zasp52*^Δ*m−/z−*^ maternal and zygotic mutant embryos. This enhancement of the *zasp52*^Δ^ zygotic phenotype supports what the co-immunoprecipitation results suggested, that Zasp52 and APC2 are likely working together to support junctions and junctional cytoskeleton involved in supracellular assemblies.

### Supracellular actomyosin cables containing Zasp52 act as mechanical insulators

Zasp52 as a component that is specifically enriched in embryonic supracellular actomyosin assemblies, in cooperation with other more widespread junctional components, appears to be important for the persistence of these cables and the morphogenetic events associated with them. It is still difficult, however, to pinpoint the collective function of such supracellular actomyosin cables during embryogenesis. Functions for certain cables have been proposed, for example for parasegmental cables in maintaining compartment identity against challenges such as cell divisions (Monier et al., 2010), or for the cable during dorsal closure in ensuring a taut and coordinated advancing front of epithelial cells (Ducuing et al., 2015). However, the function of the cable around the salivary gland placode, the cables at the ventral midline, as well as the network of ventral anterior cables present during head involution, have not yet been elucidated.

**Fig. 7.**
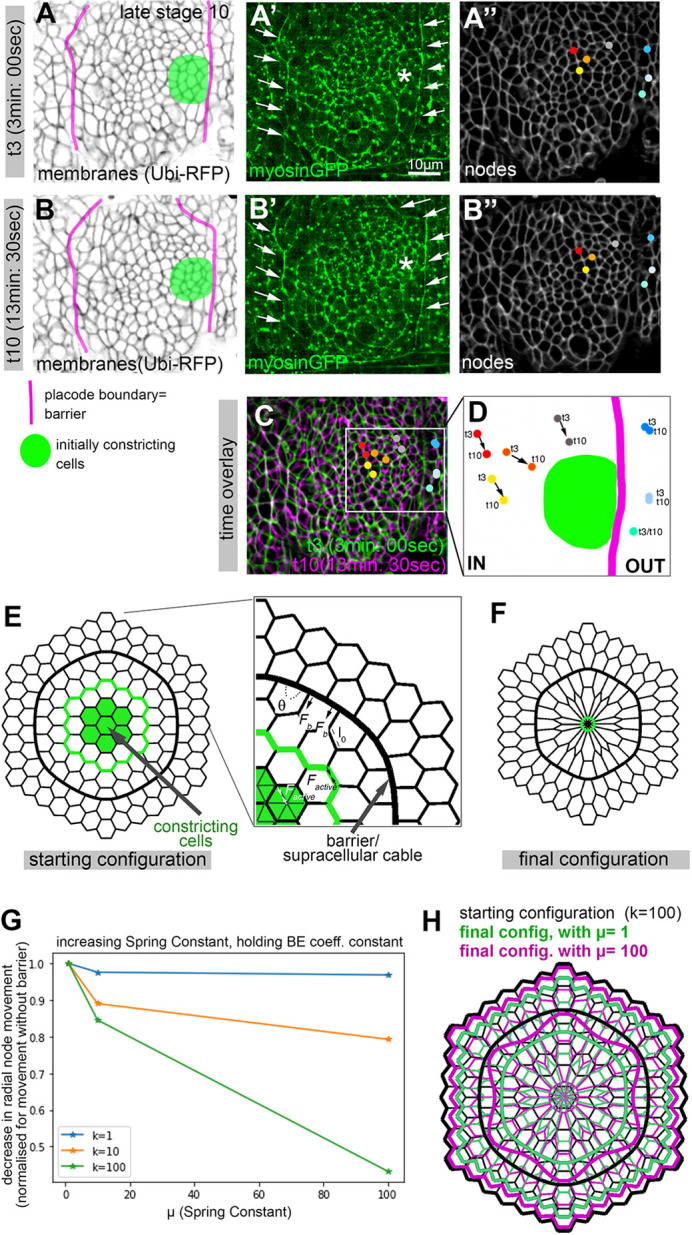
***In vivo* analysis and *in silico* modelling of the effect of a rigidity and contractility barrier on cell vertex movement.** (A-D) Qualitative analysis of cell vertex movement of vertices near the forming invagination pit where apices constrict, located inside or outside the actomyosin cable. Time-lapse movies were collected of embryos expressing a ubiquitous membrane-tethered RFP (*Ubi-RFP*) as well as a GFP-myosin regulatory light chain (*sqhGFP*) transgene (Movies 1-3). (A,B) Stills from a time-lapse movie, 10 min:30 s apart (t3 and t10 of Movie 1) with the placodal boundary actomyosin cable marked in magenta and the initial group of constricting cells at late stage 10 marked in green. (A′,B′) Stills of the same movie showing SqhGFP to show myosin. Arrows point to the position of the cable; asterisks show the position of the invagination point. (A″,B″) Vertices inside and outside the actomyosin cable near the constricting zone are marked by coloured dots at t3 (A″) and t10 (B″). (C) Both time points false-coloured and superimposed (t3 in green and t10 in magenta, with individual vertices highlighted for both timepoints). (D) Close-up of the positions of vertices inside and outside the cable at t3 and t10. The cable is marked in magenta and the constricting cells in green. (E-H) A vertex model of a simplified symmetrical 2D version of the placode was implemented similar to Durney and Feng (2021). (E) The starting configuration assumes individual cells as hexagons, apart from cells next to a barrier or supracellular cable (thick black line) where, due to an implemented contractility and hence rigidity, as well as a bending energy, cell junctions of the barrier are aligned (as is seen *in vivo*) with tricellular junctions showing 90°, 90°, 180° angles. A central group of seven cells as well as some selected edges (green) constrict to represent the forming invagination pit, and movement of nodes outside the barrier (representing the epidermis surrounding the salivary gland placode) is assessed. (F) An example of a final configuration. (G) Decrease in radial movement for the outside nodes, normalised by the movement without the barrier, for different values of a bending energy coefficient k (that reflects a penalty for the nodal angles deviating from 120° at the boundary) observed for increasing spring constants, µ. (H) A comparison of the starting configuration compared with two different final configurations, with k=100 for both and µ=1 (green configuration) or µ=100 (magenta configuration). Scale bars: 10 µm. See Fig. S6.

We suspect that one function of supracellular actomyosin cables is to insulate certain morphogenetic processes from others occurring nearby, in order to prevent undue physical interference between different morphogenetic events. Such an effect could in fact be observed when we analysed the movement of cell nodes or vertices in the early salivary gland placode, when apical constriction at the position of the future pit is only just beginning at late stage 10 (Fig. 7A-D; Fig. S6). At this stage the actomyosin cable near the forming pit at the dorsal-posterior boundary of the placode is already visible (Fig. 7A-B′; Fig. S6A-B′,D-E′). Time-lapse movies were collected for embryos expressing a ubiquitous membrane-tethered RFP (*Ubi-RFP*) as well as a GFP-myosin regulatory light chain (*sqhGFP*) transgene (Movies 1-3). Cell vertices that are inside the placode and near, but not part of, the forming invagination pit move towards the pit by about 2 μm over 10 min of apical constriction at the future pit position (Fig. 7A″,B″,C,D; Fig. S6; vertices of actively constricting cells marked in green in A and B were excluded from the analysis as these moved due to the apical constriction). By contrast, cell vertices equally close to the forming pit but posterior to the actomyosin cable, and hence outside the placode, barely move at all (Fig. 7A″,B″,C,D; Fig. S6). These observations indicate that one function of the actomyosin cable surrounding the salivary gland placode might be to serve as a barrier to insulate morphogenetic movements within the placode from the surrounding epidermis.

We set out to explore whether ‘morphogenetic insulation’ could be a general role of actomyosin cables containing Zasp52 and, in particular, what properties of such cables could confer such functionality. We already knew that the cable surrounding the salivary gland placode is under increased tension (Röper, 2012). We now employed a simplified *in silico* 2D vertex model of the salivary gland placode to test whether a tensed boundary could behave as a morphogenetic insulator (Fig. 7; Durney and Feng, 2021). This model considers a centre of hexagonal cells surrounded by a barrier of increased contractility and stiffness to model the actomyosin cable surrounding the placodal cells, with a further two coronae of cells surrounding the barrier that represent the surrounding epidermis (Fig. 7E). In this model, the central cells constrict, thereby pulling on and deforming other cells on the inside and outside of the barrier (Fig. 7F). The starting configuration takes into account that junctions at the barrier *in vivo* are more aligned and hence vertices at tricellular junctions deviate drastically from 120°. This is reflected in the model by the contribution of a bending energy coefficient (k) on edges representing the supracellular barrier. The central cells are allowed to actively constrict and the average nodal movement towards the centre is measured, while the spring constant (µ) of the barrier is varied. For a set value of the bending energy coefficient k (1, 10 or 100) an increase in spring constant µ consistently led to a decrease in the nodal movement of cells outside the barrier towards the centre (Fig. 7G,H), highly reminiscent of the reduced or absent movement of nodes we observed *in vivo*.

Thus, the *in vivo* and *in silico* data suggest that during mid to late embryogenesis in the fly the many large-scale actomyosin cables observed could act as insulators, thus ensuring that morphogenetic movements do not spread beyond a defined primordium, or act to prevent neighbouring regions undergoing different morphogenetic processes from unduly influencing each other.

## DISCUSSION

The formation of organs during development requires coordination at many different time and length scales. Cell behaviours within a tissue primordium need to be coordinated but, equally, at boundaries between differently fated embryonic regions or organ primordia, such coordination has to cease so as not to inadvertently affect the neighbouring processes. Coordination between groups of cells is often achieved through coordination of the cell cytoskeletal systems, via alignment of actin or microtubules, and is transmitted through cell-cell adhesion complexes usually located at adherens junctions (Röper, 2013; Sánchez-Corrales and Röper, 2018).

An intriguing cytoskeletal coordination is observed during numerous morphogenetic events in both invertebrates and vertebrates in the seemingly supracellular arrangement of actomyosin into supracellular cables. In *Drosophila*, numerous actomyosin cables are formed throughout embryogenesis, from early parasegmental cables and cables flanking the midline to cables surrounding and connecting tissue primordia at later stages. Though some studies have offered glimpses of functions or mechanisms of assembly for particular cables (Hashimoto and Munro, 2019; Major and Irvine, 2006; Monier et al., 2010; Paré et al., 2019, 2014; Röper, 2012; Sidor et al., 2020), major questions about their assembly, composition and general function remain. Though seemingly supracellular, individual cable segments in cells are of course connected to neighbouring parts at cell-cell junctions. But are these junctions in any way different to regular adherens junctions? Furthermore, are there specific components of such supracellular cables that set them apart from ubiquitous cortical actomyosin?

Zasp52 as a core component of Z-lines in muscles is the first component of supracellular actomyosin cables that distinguishes these from regular junctional actomyosin. We identified an actin-binding-motif in the Zasp52 central domain, as well as a host of junctional proteins interactors, and furthermore its N-terminus, known for binding actin (Liao et al., 2020), also promotes Zasp52 dimerisation and multimerisation. These results suggest that, upon binding to Zasp52, junctional actomyosin likely takes on a new and higher-order structure. It is noteworthy that the junctional interaction partners localise to the marginal zone (Crumbs, Patj), the adherens junctions (armadillo, α-Catenin, p120-Catenin, E-Cadherin, APC2, Sidekick, Bazooka, Polychaetoid, Canoe) and even basally to adherens junctions (Scribble). This suggests that the increased size of actomyosin cables, which can be inferred visually from the strongly increased intensity of F-actin and myosin labelling in these structures, means that they span an enlarged region along the lateral membrane compared with general junctional actomyosin that is usually restricted to adherens junctions only (Desai et al., 2013; Röper, 2015). Such lateral expansion of actomyosin accumulation could be a defining feature of supracellular actomyosin cables, with a possible actomyosin organisation across the epithelial junctions similar to lateral stacking of thin filaments in sarcomeres, though this requires future investigation.

The phenotypes observed in the absence of Zasp52, as well as in the combined absence of Zasp52 and APC2 (but not APC2 alone), in particular the epidermal tears and holes, are reminiscent of phenotypes observed when adherens junctions and their linkage to the actin cytoskeleton are compromised by the lack of α-Catenin (Sarpal et al., 2012), or by hypomorphic mutations in E-Cadherin itself (Tepass et al., 1996). These mutant embryos also show epidermal holes, especially in the head and ventral region, as well as anterior disorganisation that might be a result of head involution defects. Thus, impairment of the linkage of adherens junctions to the junctional actin cytoskeleton, occurring in α-Catenin, E-Cadherin and also Zasp52 mutants, appears to lead to a common set of phenotypes.

Interestingly, several of the junctional components identified in the complex with Zasp52 are either enriched at or are exclusive to tricellular junctions (APC2, Canoe, Sidekick), and Zasp52 is enriched in these tricellular junctions itself. These cell vertices are the points at which individual segments of actomyosin cables require to be connected to the next cable segment one cell on. The presence of Zasp52 in precisely these junctions could help to reinforce them against the increased tension that actomyosin cables usually exert and bear (Fernandez-Gonzalez et al., 2009; Röper, 2012).

Recruitment of Zasp52 to a forming cable might well occur through a positive feedback loop. Initial Zasp52 recruitment to increased junctional F-actin at a position where a cable is forming, for example at the boundary of the placode (downstream of Crumbs anisotropy and Rok accumulation; Röper, 2012; Sidor et al., 2020), could structurally change the cable. This could be via strengthening it through actin crosslinking, or via strengthening and amplifying the connection to junctions, which in turn could promote further actin and myosin recruitment, as well as increased recruitment of Zasp52 itself. Actomyosin enrichment, for example in the salivary gland placodal cable, occurs before Zasp52 recruitment to the same location. Thus, it appears to be the localisation of actomyosin, not of Zasp52, that determines where a cable forms. Rather, actomyosin cables containing Zasp52 might assemble at the coincidence of two events: first, the enhanced accumulation of junctional actomyosin triggered by other mechanisms, and second, the expression of Zasp52 in the very same cells, thereby leading to its recruitment via interactions with junctional binding partners to the forming cable. This recruitment then adds additional cable characteristics. The role of Zasp52 in altering the assembly or structure of a cable could be even more profound, as its role in Z-lines of sarcomeres could suggest that actomyosin cables might have a higher order of actin and myosin assembly than general actomyosin, imposed by recruitment of Zasp52. Such higher order was observed previously in specialised junctions of support cells in the mouse cochlea, as a feature of fully differentiated cells rather than a tool of morphogenesis (Ebrahim et al., 2013). The loss of actin accumulation at cable sites in the *zasp52* mutant embryos strongly suggests that actin stabilisation could be a key part of Zasp52's function. Dissecting these structural changes in detail will be the next challenge in understanding actomyosin cable function.

Can our analysis of the phenotypes observed in *zasp52* mutants furthermore allow us to identify a more general role for actomyosin cables during morphogenesis? One key role could be that the higher stiffness and contractility of actomyosin cables allows them to serve as physical barriers, thereby insulating morphogenetic events and preventing them from unduly influencing surrounding tissues. Such undue spread of movement can easily be imagined as all epithelial cells will be mechanically coupled to neighbouring cells to some degree due to their epithelial junctions. Hence, defined mechanical barriers could be an important requirement during epithelial morphogenesis. To assess this option, we turned to the properties of the actomyosin cable surrounding the salivary gland placode. Our combined *in vivo* and *in silico* approaches demonstrate that the placode cable is likely acting as a mechanical insulator, which could therefore also be true for other cables. A further role could be that the presence of interlinked cables spanning large regions of the epidermis allows a coordination of large-scale movements of different tissue primordia. In the *Drosophila* embryo, this might well be the case for the interconnected cables in the ventral head region. And matching this, the *zasp52* complete null mutant shows major defects in this process. More detailed whole embryo studies in the future will reveal how common these functions for actomyosin cables are.

In summary, the analysis of Zasp52 as a component of actomyosin cables has revealed what we suspect are key functions of actomyosin cables in epithelial morphogenesis in animals: the mechanical insulation of individual morphogenetic events as well as the coordination of large-scale epidermal changes. It will be interesting to analyse whether these functions of Alp/Enigma proteins are conserved in other prevalent cables observed during vertebrate morphogenesis.

## MATERIALS AND METHODS

### Fly husbandry and genetics

*Drosophila melanogaster* embryos were collected for staining on apple juice agar plates at 25°C overnight or for durations as indicated. Embryos used for live imaging were collected at 25°C and aged 2 h at 29°C before imaging. Embryos for immunoprecipitations were pre-laid on 90 mm apple juice agar plates for 1 h to avoid collecting older retained embryos, the plate was discarded and collections started for 9, 10, 11 or 12 h. Overnight collections without pre-lay were collected in the same manner. SeeTable S3for key resources, and Table S4 for genotypes used in figure panels.

### Generation of zasp52^Δ^ germline clones

To generate embryos devoid of maternally deposited Zasp52, the FLP-DFS strategy was employed, based on the work of Chou and Perrimon (1992). To this end, *FRT G13 zasp52*^Δ^ virgins were crossed to males of *hsFLP; FRT G13 ovoD1/TTP* stock. Progeny of this cross was heat shocked for 1 h at 37°C when reaching first instar stage and again the following day. From these crosses, virgin females were picked and crossed to *zasp52*^Δ^*/CyO twi::GFP* males and embryos collected and analysed.

### Immunofluorescence staining of whole-mount embryos

Embryos of desired stages were collected from apple juice-agar plates with a brush and tap water and transferred into a sieve. After rinsing with tap water, embryos were dechorionated with 50% of thick bleach/water for 3 min. Embryos were washed several times with water to remove bleach solution and residual water absorbed with paper wipes. Embryos were then taken up in 800 µl heptane and placed in a small glass screw-cap vial. Then 400 µl PBS and 400 µl 8% paraformaldehyde (PFA; EM-grade)/PBS were added to the embryos and embryos left to fix on a shaker for 15 min. The lower phase containing PFA was removed and embryos devitellinised in 1 ml 90% ethanol/H_2_O with shaking. The embryos were stored at −20°C in ethanol.

Devitellinised and fixed embryos were simultaneously permeabilised and blocked with PBT [0.3% Triton X-100, 2.5% bovine serum albumin (BSA) in PBS] for 1-3 h at 4°C. Primary antibodies were diluted in PBT and embryos incubated with antibodies overnight at 4°C on a shaker. Embryos were washed three times with 500 µl PBT for 20 min on shaker at room temperature (RT). Secondary antibodies were diluted 1:200 in PBT or according to the manufacturer and added to washed embryos to incubate for 2.5 h at RT or overnight at 4°C. Embryos were subsequently washed 3× with PBT for 20 min on a shaker at RT. Embryos were mounted in Vectashield mounting medium (H-1000).

### Live imaging of whole-mount embryos

For live time-lapse experiments, embryos of the genotype *sqh^AX3^;sqhGFP; UbiRFP* were dechorionated in 50% bleach and extensively rinsed in water. Stage 10 embryos were manually aligned and attached to heptane-glue-coated coverslips and mounted on custom-made metal slides; embryos were covered using halocarbon oil 27 (Sigma-Aldrich) and viability after imaging after 24 h was controlled prior to further data analysis. Time-lapse sequences were imaged under a 40×/1.3NA oil objective on an inverted Zeiss 780 Laser scanning system, acquiring *z*-stacks every 0.58-3 min with a typical voxel *xyz* size of 0.22×0.22×1 μm. *Z*-stack projections to generate movies were assembled in ImageJ or Imaris. The membrane channel images from time-lapse experiments were denoised using *nd-safir* software (Boulanger et al., 2010).

### Ectopic expression of Zasp52 in engrailed stripes

Zasp52 truncations were expressed with the UAS-GAL4 system using the *enGal4* driver. To this end, virgins of *+/+; enGAL4/CyO, UAS-GFP* were crossed with w*/Y; UAS-Zasp52-PK-6xHis-FLAG/TM3 twi::GFP or w*/Y; +/+; UAS-Zasp52-PRΔ*PDZ-FLAG/TM3 twi::GFP*, and the resulting F1 embryos fixed and stained with anti-Cadherin and anti-FLAG antibodies as well as with Rhodamine-Phalloidin. For further details of antibodies and reagents see Table S3. Fluorescent images of engrailed stripes in the ectoderm were acquired using an Olympus FluoView 1200.

### Quantification of F-actin signal intensity at the supracellular actomyosin cable in the placode

Whole-mount embryos were fixed and stained as described above. Higher resolution images of placodes were acquired as *z*-stacks using the Olympus FluoView 1200 microscope. Equivalent slices (eight slices corresponding to 400 nm, starting apically at first-observed intensity of E-Cadherin) were used to create a SUM projection. Three rectangular areas were defined over the whole placode to measure background intensity and calculate average background intensity. Phalloidin signal intensity of individual junctions at the placode border was measured, leaving out the two rows of cells at the ventral midline and the junctions closest to the invagination pit because of distortion of the tissue at these points. Junction intensity was normalised to background [(I_Junction_/Area_Junction_)/(I_bkgd_/Area_bkgd_)].

### Transfection and staining of S2 cells with Zasp52 truncations

S2 cells (University of California, San Francisco, USA; mycoplasma-free judged by DAPI staining) were grown at 25°C in Schneider's Medium (Thermo Fisher Scientific) supplemented with 1% Pen/Strep (Gibco) and 10% (vol/vol) fetal bovine serum (Gibco; heat-inactivated for 1 h at 70°C). Stable cell lines were obtained by transfection with pMT puro vectors containing either Zasp52-PF or Zasp52-PF-ABM using Effectene (Qiagen), according to the manufacturer's instructions, followed by selection in 5 µg/ml Puromycin (Thermo Fisher Scientific). Expression was induced with 0.6 mM CuSO_4_ (in selection medium) for 2 days. Ibidi chambers were coated with 50 µg/ml Concanavalin A (diluted in PBS) for 10 min and washed with PBS. Cells were added in medium to the wells and allowed to spread for 1 h. Cells were washed with PBS, fixed with 4% PFA (diluted in PBS) for 20 min, permeabilised with Triton X-100 (0.1% in PBS), washed and stained with primary antibody (diluted in 0.1% BSA-PBS) for 1 h. Cells were washed with PBS and stained with secondary antibody (diluted in 0.1% BSA-PBS) for 1 h, washed and imaged directly in PBS. Images were acquired using a Zeiss 880 confocal microscope.

### Recombinant expression and purification of the central Zasp52 ABM

The Zasp52 ABM was expressed as a GST fusion protein in *Escherichia coli* and purified via affinity and size exclusion chromatography. The plasmid was generated via synthetic assembly of aa1131–aa1318 of the Zasp52-PF cDNA (synthetic assembly of Zasp52-PF cDNA, Twist Bioscience) with AscI and FseI restriction enzyme overhangs and was digested and ligated in pGEX F/A vector cut with AscI and FseI. The pGEX plasmids containing truncations were transformed into the Rosetta (DE3) strain via heat shock transformation and selected via antibiotic selection. Colonies were picked the next day to inoculate pre-cultures. Precultures were used to set-up large cultures grown to OD_600_=0.6 and expression was induced by addition of 0.5 mM IPTG. Cultures were grown overnight at 18°C and harvested the next day via centrifugation at 4000 ***g*** for 30 min. Supernatant was discarded and pellets lysed on ice by resuspending in lysis buffer [50 mM Tris-HCl (pH 7.4), 150 mM KCl, 1% Triton X-100, 10 mM MgCl_2_, 5% glycerol, 1 mM DTT, 1× complete protease inhibitors, 0.7 mg/ml lysozyme and 10.00 µg/ml DNAse I in water] and then sonicated on ice, followed by clearing of lysate via centrifugation at 60,000 ***g*** for 30 min at 8°C. Protein-containing supernatant was incubated with 1-5 ml of GSH-beads equilibrated in lysis buffer on a shaker for 2 h at 4°C. Flow-through was collected and the column was washed once with 45 column volume (CV) of wash buffer [50 mM Tris-HCl (pH 7.4), 150 mM KCl, 5% glycerol, 1 mM DTT]. To remove chaperones, an additional wash step with 1 CV ATP wash buffer [50 mM Tris-HCl (pH 7.4), 150 mM KCl, 5% glycerol, 10 mM NaATP, 10 mM MgCl_2_, 1 mM DTT] was carried out. GST-fusions were eluted with 10 mM glutathione [100 mM Tris-HCl (pH 7.4), 150 mM KCl, 10 mM GSH, 5% glycerol, 1 mM DTT] in 1 CV fractions. Fractions rich in GST-fusion protein were pooled and cleaved using TEV enzyme during dialysis [50 mM Tris-HCl (pH 7.4), 150 mM KCl, 5% glycerol, 1 mM DTT] overnight at 4°C. In order to remove free GST from the solution, the dialysed protein was incubated with equilibrated GSH resin for 2 h at 4°C and the flow through collected, concentrated and injected in a Superdex 200 (16/100, GE Healthcare) exclusion chromatography column. The column was equilibrated with gel filtration buffer [20 mM K-HEPES (pH 7.5), 150 mM KCl, 5% glycerol, 1 mM DTT]. Fractions collected were analysed by staining on SDS PAGE and fractions chosen, pooled and concentrated. Aliquots were frozen in liquid nitrogen and stored at −80°C until used for experiments. See Table S5 for the DNA sequences of cloned Zasp52 fragments or isoforms.

### F-Actin binding assay

To investigate protein binding to filamentous actin, an F-actin pelleting assay was employed. To this end, rabbit muscle actin (Akl99, Cytoskeleton) was prepared according to the manufacturer's instruction and Ca^2+^-actin exchanged to Mg^2+^-actin with exchange buffer (0.2 mM EGTA, 0.02 mM MgCl_2_ final) in G-buffer [5 mM Tris-HCl (pH 8.0), 0.2 mM ATP, 0.1 mM CaCl_2_, 0.5 mM DTT] on ice for 10 min. Mg^2+^-G-actin stock solution was diluted to 8 µM, 6 µM, 4 µM and 2 µM and co-incubated with Zasp52-ABM fragments at 8 µM in 1× polymerisation buffer [50 mM KCl, 1 mM MgCl_2_, 1 mM EGTA, Imidazole-HCl (pH 7.0)] for 1 h at RT in ultracentrifugation tubes. The samples were then centrifuged at 200,000 ***g*** for 1 h at 4°C. Then, 90% of the supernatant was decanted and the pellet was resuspended in the same volume of sample buffer. Supernatants and pellets were analysed via SDS PAGE and stained with Instant Blue and colorimetric image recorded with ChemiDoc XRS+ (Bio-Rad). Band intensity was quantified by densitometric scanning using Image Lab software, background intensity subtracted and ABM fraction binding quantified [Int(ABM_bound_)/Int(ABM_bound_)+Int(ABM_unbound_)].

### Immunoprecipitation of GFP-labelled proteins from *Drosophila* embryos

To identify proteins associated with Zasp52 in epithelia, co-immunoprecipitations against GFP were performed using either the *Zasp52-GFP[ZCL423]* or *Zasp52-GFP[G00189]* endogenous protein trap lines as well as wild-type *yw* flies (Morin et al., 2001). *Armadillo-YFP[CPTI001198]* was used as a control for co-immunoprecipitation of a junctional cytoplasmic protein. To prevent the contamination of the sample with muscle tissue, the collection protocol consisted of a 1 h pre-lay collection to eliminate embryos retained in the mother (discarded), and was then followed by a 9 h collection, resulting in embryos between 0 and 9 hpf and thus prior to muscle development, which commences at 9:20 hpf. Dechorionated frozen embryos were placed on ice and immediately covered with 100 µl chilled lysis buffer [50 mM Tris (pH 7.4), 150 mM KCl, 0.5 mM EDTA, 0.1% Glycerol, 0.01% Triton X-100, 1200 μg/ml benzamidine, 40 μg/ml chymostatin, 40 μg/ml antipain, 2 μg/ml leupeptin, 0.96 μg/ml pefabloc, 0.5 mM PMSF]. Each sample contained roughly the same mass of embryos, as different samples were used per experiment in order to observe differences between sample types, e.g. wild-type versus Zasp52-GFP, and quantitative analysis of triplicate samples used. If necessary, different collections of equivalent laying conditions were pooled to create one sample. Per sample, 500 µl of lysis buffer were added and total sample transferred into a glass dounce-homogeniser. Embryos were lysed with 20 strokes and a subsequent 30 min incubation on ice. To separate the protein-containing aqueous phase from lipids and cell debris, samples were centrifuged at maximum speed in a tabletop centrifuge for 10 min at 4°C. The aqueous phase was carefully collected and added to equal amounts of GFP-nanobody coated magnetic beads (Chromotek), equilibrated in lysis buffer. Samples were incubated on a rotating shaker for 1.5 h at 4°C. Samples were then washed 3× with wash buffer [10 mM Tris (pH 7.4), 150 mM KCl, 0.5 mM EDTA, 0.1% Glycerol, 0.5 mM PMSF] and then either eluted in sample buffer for subsequent analysis by western blotting, or the wash buffer decanted, beads resuspended in 50 mM ammonium bicarbonate and stored at −20°C before mass spectrometry analysis.

### Mass-spectrometry processing of immunoprecipitation samples

Proteins were prepared for enzymatic cleavage from magnetic beads by submersion in 50 mM ammonium hydrogen carbonate (pH 8.0). The solution containing bead-bound protein was digested with 0.5 µg of trypsin for 60 min at 37°C in a thermomixer, shaking at 800 rpm. This was followed by another overnight digestion step for which an additional 1 µg trypsin was added and the samples digested under the same conditions as above. The reaction was terminated by adding formic acid to a final concentration of 2% v/v. The output of the digestion reaction was analysed using a nano-scale capillary liquid chromatography with tandem mass spectrometry (LC-MS/MS) in an Ultimate U3000 HPLC setup (Thermo Fisher Scientific Dionex) to deliver a flow of ∼300 nl/min. A C18 Acclaim PepMap100 5 µm, 100 µm×20 mm nanoViper (Thermo Fisher Scientific Dionex), trapped the peptides before separation on a C18 Acclaim PepMap100 3 µm, 75 µm×150 mm nanoViper (Thermo Fisher Scientific Dionex). The Peptides were eluted from the column via an acetonitrile gradient. The analytical column outlet was immediately interfaced by a modified nano-flow electrospray ionisation source, coupled to a hybrid dual pressure linear ion trap mass spectrometer (Orbitrap Velos, Thermo Fisher Scientific). The data acquisition was carried out using a resolution of 30,000 for the full MS spectrum, then followed by ten MS/MS spectra in the linear ion trap. All MS spectra were collected over an m/z range of 300-2000 and MS/MS scans were collected with a threshold energy of 35 for collision-induced dissociation. All raw files were processed with MaxQuant 1.5.5.1 (Cox and Mann, 2008) using standard settings and searched against the UniProt KB with the Andromeda search engine (Cox et al., 2011) integrated into the MaxQuant software suite. Enzyme search specificity was Trypsin/P for both endoproteinases. One or two missed cleavages for each peptide were allowed. Carbamido-methylation of cysteines was selected as fixed modification with oxidised methionine and protein N-acetylation set as variable modifications. The search was conducted with an initial mass tolerance of 6 ppm for the precursor ion and 0.5 Da for MS/MS spectra. The false discovery rate was fixed at 1% at the peptide and protein level.

### Quantitative analysis of mass-spectrometric data of co-immunoprecipitations in Perseus

Downstream statistical analysis was carried out using the Perseus interface of MaxQuant (https://maxquant.net/perseus/). Prior to statistical analysis, peptides mapped to known contaminants, e.g. human keratin, reverse sequence hits and protein groups only identified by site, were removed. Only protein groups identified with at least two peptides, of which one had to be unique, and two quantitation events were considered for data analysis with Perseus. Three experiments were analysed for Zasp52-GFP and Armadillo-YFP each and two for wild type. To compare samples of interest with control, the triplicate or duplicate datasets were transformed to Log2(x), missing values replaced by imputation, and the volcano plot tool used to compare two datasets in which the *x*-axis represents a difference score and the *y*-axis visualises the negative, logarithmic *P*-value generated by an unpaired two-tailed Student's *t*-test. The significance threshold was set to a *P*-value of 0.75, so that known interaction partners in the Armadillo-YFP control sample were identified.

### Computational modelling

We represent an epithelial tissue by a 2D vertex model of 127 hexagonal cells initially arranged in a hexagonal orientation, essentially following previous reports (Durney and Feng, 2021; Durney et al., 2018). In brief, an individual cell has six peripheral nodes and one central node connected by passively elastic edges. To represent a supracellular actomyosin cable that is enriched by myosin across several junctional boundaries, we have taken select edges (indicated in bold black, Fig. 7E,F) and endowed them with two different properties: an increased elastic modulus, μ, and a resistance to bending. The latter is implemented by a penalty against deviation in the angle of adjacent edges resulting in a restoring force, ***F***_*b*_. Select edges (indicated in green, Fig. 7E) are prescribed to contract and generate active pulling forces. This contraction pulls on the tissue exterior of the boundary, compressing the interior region.

The total force, ***F***_*i*_, on node *i* is given by,

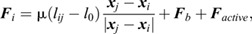
where *l*_*ij*_ is the current edge length between nodes *i* and *j*, *l*_0_ is the edge rest length, ***F***_*b*_ is the force due to bending (only on edges associated with the supracellular actomyosin cable) and ***F***_*active*_ is the active force that causes contraction of select edges. Nodal motion is governed by over-damped dynamics with a viscous friction factor η,

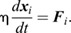
The force, *F*_*b*_=*k*(θ−π), tends to align the adjacent edges. As the tissue is in an initial hexagonal configuration, this term circularises the supracellular cable and reduces its circumference. To maintain a zero-energy ground state, we set the bending modulus, *k*, and then allow the tissue to relax to equilibrium. For edges of the supracellular cable, we adopt the equilibrium length to be its new rest length. This procedure is repeated until there are no more changes in 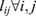. This guarantees a consistent ground state as μ is increased. Tissue deformation is initiated by *F*_*active*_ contracting specific edges.

The main output of the model is the average radial movement of the *n* peripheral nodes of the epithelial tissue,

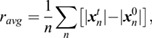
where the superscript denotes simulation time.

## Supplementary Material

10.1242/develop.201238_sup1 Supplementary information
