# Supplementary material for: Zasp52 strengthens whole embryo tissue integrity through supracellular actomyosin networks
Source: Development. 2023 Apr 3;150(7):dev201238. doi: 10.1242/dev.201238 (PMC10112930; doi:10.1242/dev.201238)
Supplement: Supplementary information [file develop-150-201238-s1.pdf]

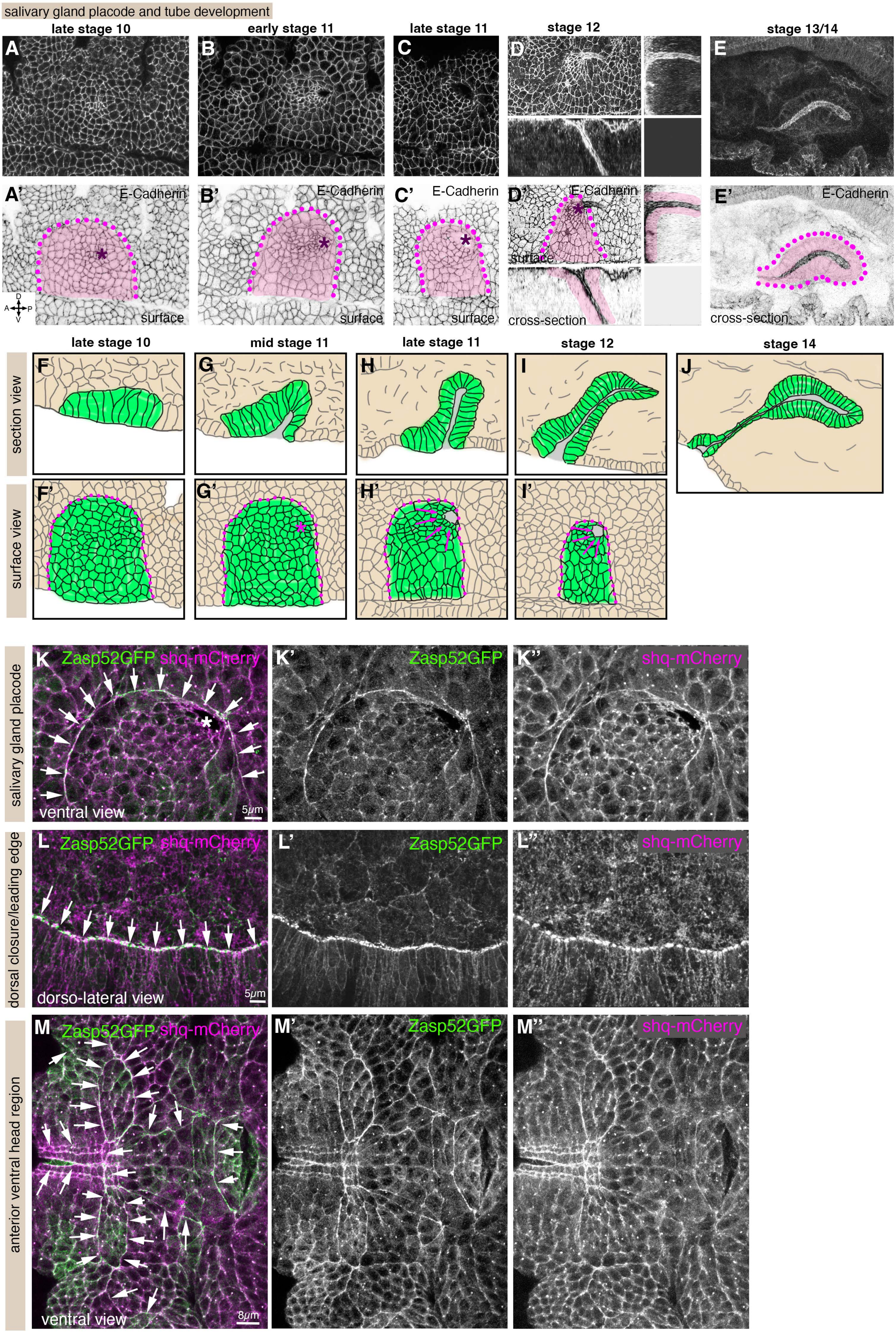


Supplemental Figure S1_Ashour et al.

**Fig. S1, related to Figures 1, 2, 4, 5 and 6. Embryonic development of the salivary gland and Zasp52 localisation in developmental actomyosin cables.**
**A-E'** Development of the salivary gland begins at late stage 10 from a flat epidermal placode of about one hundred cells each on either side of the ventral midline (**A, A'**). **B-C'** Cells invaginate through a focal point in the dorsal-posterior corner, marked by asterisks. **D-E'** A narrow-lumen tube is formed as soon as cells internalise and the tube extends internally with more cells invaginating. Purple dotted lines mark the boundary of the placode, pink overlay indicates the salivary gland cells, and asterisks mark the position of the invagination point or pit. Surface views (**A-C'** and main panels in **D**, **D'**) and cross section views (small panels in **D**, **D'**; and **E**, **E'**) are shown. E-Cadherin labelling of apical junctional cell outlines is shown, with lower panels showing inverse label to better visualise labels.
**F-I'** Schematic matching section views (**F-J**) and surface views (**F'-I'**) of salivary gland development and invagination from late stage 10 until all cells are internalised at stage 14, saluvary gland fated cells are in green, the surrounding tissue in beige. Magenta dots in **F'-I'** mark the position of the actomyosin cable at  the placode boundary. The asterisk in G' marks the invagination point, arrows in **H'** and **I'** illustrate the movement of cells towards the invagination point.
**K-M''** Zasp52 localisation in actomyosin cables during embryonic development in comparison to Sqh (non-muscle myosin II regulatory light chain). Zasp52-GFP-Z is in green, Sqh-mCherry in magenta. **K-K''** shows the cable at the boundary of the salivary gland placode, **L-L''** the cable during dorsal closure at the leading edge-amnioserosa interface, **M-M''** the network of anterior ventral cables during head involution. Arrows point to the position of the cables.

Development • Supplementary information

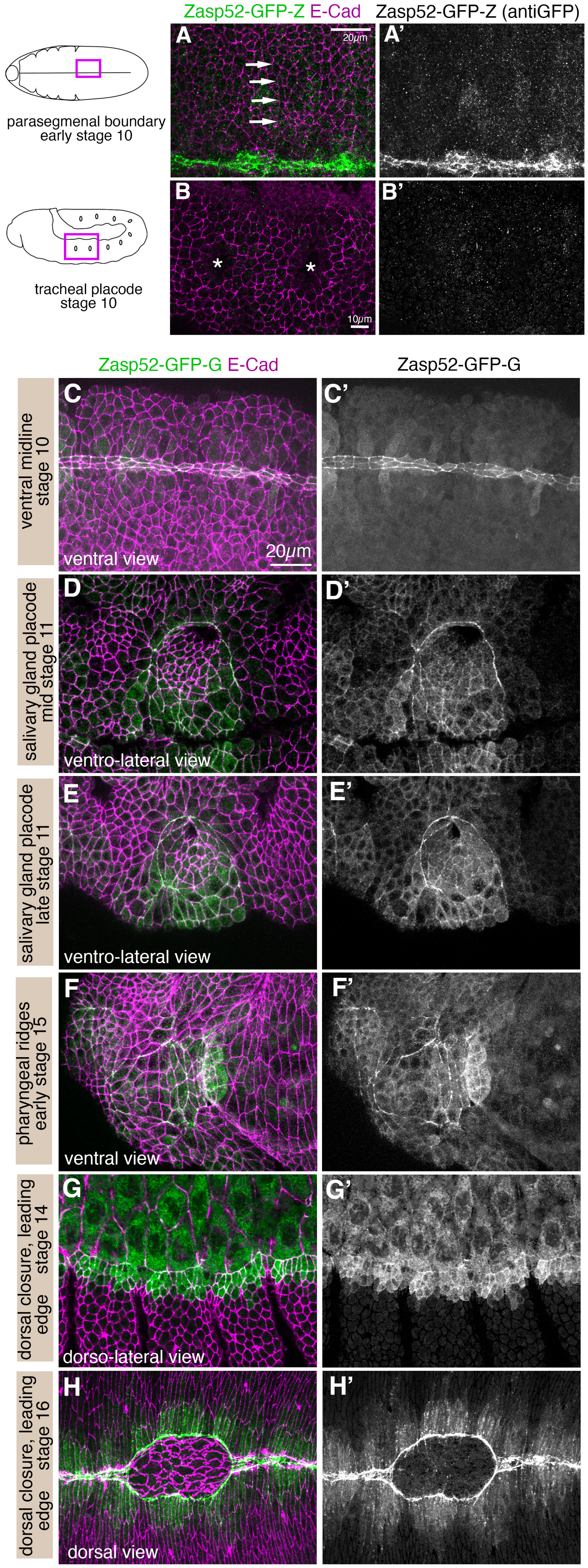


Supplemental Figure S2_Ashour et al.

**Fig. S2, related to Figure 1. Zasp52-GFP is not found in parasegmental or tracheal pit actomyosin cables and localisation of Zasp52-GFP-G*.* A**, **A'** A parasegmental boundary within the embryonic epidermis at early stage 10. Note that Zasp52-GFP in green in **A** and as a single channel in **A'** is strongly expressed in the ventral midline but not at all enriched at parasegmental boundaries (arrows in **A**) at this stage.

**B**, **B'** Two tracheal placodes within the embryonic epidermis at stage 10, the invagination points are marked by asterisks. Zasp52-GFP is not enriched in any junctions within these placodes.

**C-H'** Localisation of the Zasp52[G00189] protein trap line in the *Drosophila* embryo, in the ventral midline at stage 10 (**C**, **C'**), salivary gland placode at mid stage 11 (**D**, **D'**) and late stage 11 (**E**, **E'**), pharyngeal ridges at early stage 15 (**F**, **F'**), the dorsal closure leading edge at stage 14 (**G**, **G'**) and the dorsal closure leading edge at stage 16 (**H**, **H'**). Zasp52-GFP-G is in green and as a single channel and E-Cadherin is in magenta.

Development • Supplementary information

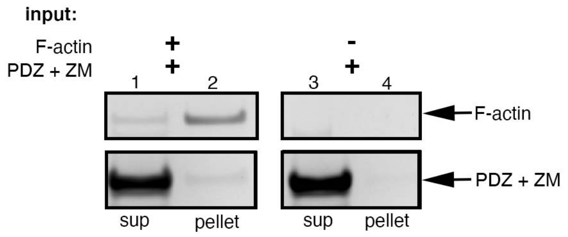


Supplemental Figure S3_Ashour et al.

**Fig. S3, related to Figure 2. F-actin binding of Zasp52 N-terminus.** The Zasp52 PDZ domain and Zasp motif were expressed in bacteria and purified protein was used in an F-actin pelleting assay. F-actin can co-sediment a fraction of PDZ+ZM (lane 2), whereas PDZ+ZM alone does not pellet (lane 4) but remains in the supernatant (lane 3).

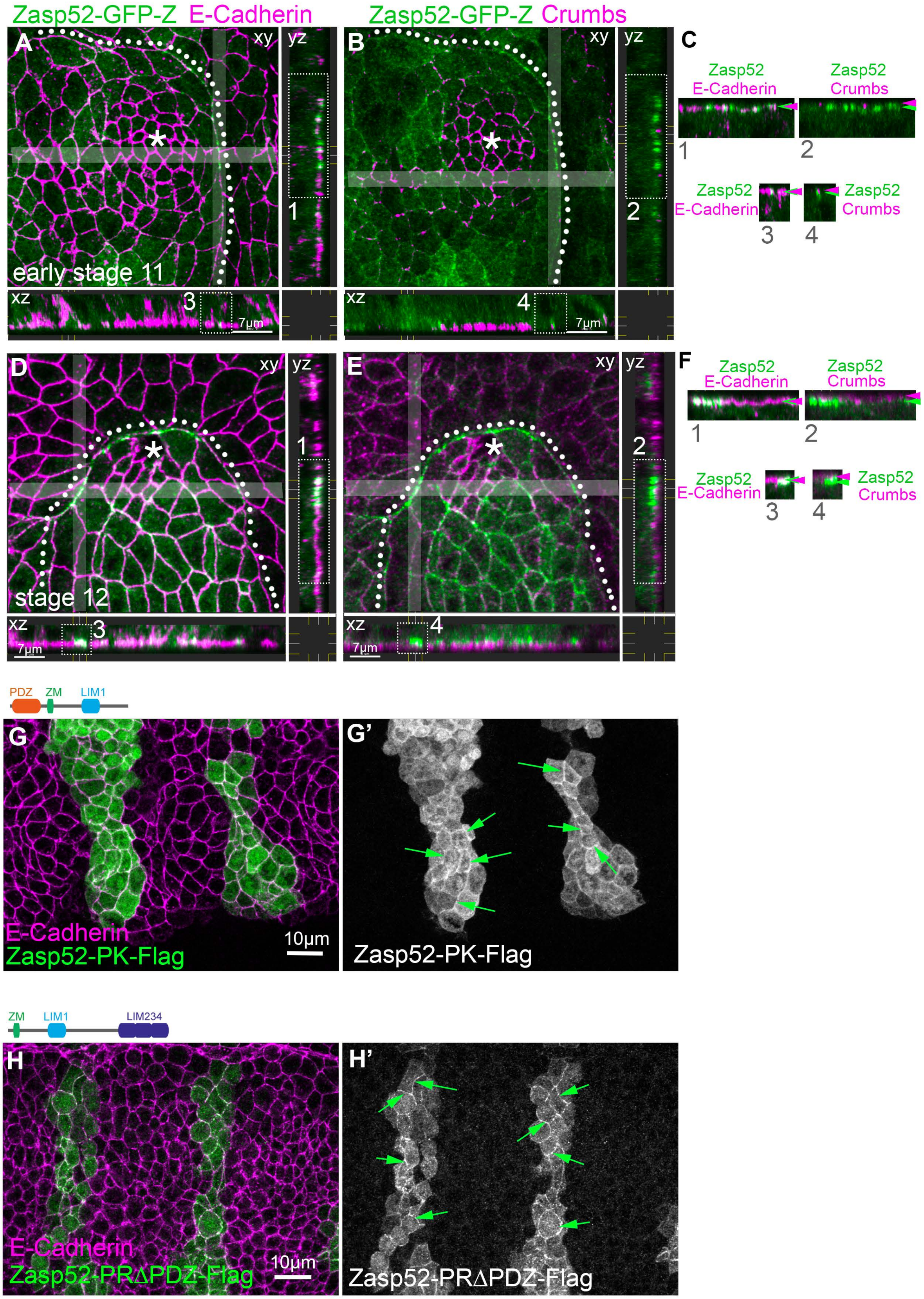


Supplemental Figure S4_Ashour et al.

**Fig. S4, related to Figure 3. Endogenous and ectopic Zasp52 localisation at cell-cell junctions.**

**A-F** Localisation of Zasp52GFP in actomyosin cables along the apical-lateral side of cells. Examples of localisation in the actomyosin cable surrounding the salivary gland placode at early stage 11 (**A**-**C**) and at stage 12 (**D**-**F**). Zasp52-GFP (green) localisation is compared to E-Cadherin at adherens junctions (magenta in **A**, **D**) and to Crumbs in the marginal zone
(magenta in **B**, **E**). Surface stack views (xy) with corresponding cross sections (xz and yz) are shown, with the width of the section displayed indicated by grey shading. White dotted boxes in the cross sections (labelled 1-4) are displayed again in **C** and **D** to compare the localisation of Zasp52-GFP, E-Cadherin and Crumbs along the apical to basal direction, apical is up in **C** and **D**. Coloured arrowheads indicate the position of Zasp52GFP (green) or E-Cadherin and Crumbs (magenta) as indicated. White dotted lines indicate the boundary of the salivary gland placodes shown. Scale bars are 7µm.

**G**, **G'** Stripe-overexpression of the Zasp52-PK isoform (as *UAS-Zasp52-PK-Flag*), containing the PDZ domain, Zasp motif and LIM1 domain, under control of *enGal4* leads to it being localised to cell-cell junctions in addition to a cytoplasmic pool.

**H**, **H'** Stripe-overexpression of the Zasp52-PRΔPDZ isoform (as *UAS-Zasp52-PRΔPDZ -Flag*), containing the ZM motif and LIM 1-4 domains, under control of *enGal4* leads to it being localised to cell-cell junctions in addition to a cytoplasmic pool.

Zasp52 isoforms are in green and as a single channel in **G'**, **H'** and E-Cadherin to label cell junctions in magenta. Arrows point to junctional localisation of the ectopically expressed Zasp52 protein variants.

Development • Supplementary information

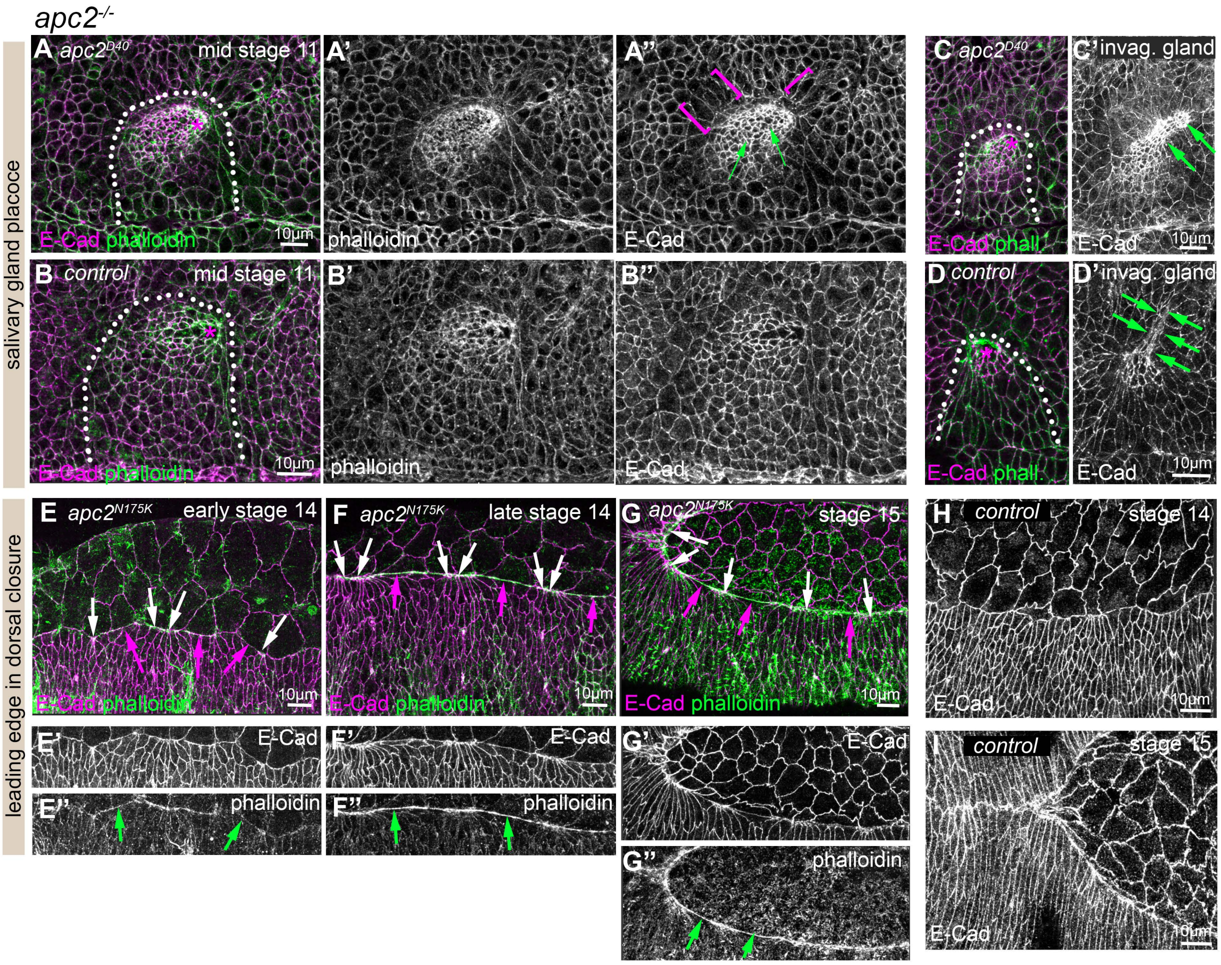


Supplemental Figure S5_Ashour et al.

**Fig. S5, related to Figure 6. apc2$^{-/-}$ mutant embryonic phenotypes. A-B'** *apc$^{D40}$* mutant salivary gland placodes (**A-A''**), at mid stage 11 when invagination has begun, display secretory cells that are more constricted than usual (**A''**, green arrows), and cells immediately surrounding the placode boundary are overstretched (**A''**, magenta brackets), compared to control placodes of the same stage (**B-B''**). **C**,**C'** The invaginated portion of the salivary gland at stage 12 in an *apc$^{D40}$* mutant embryo shows a too wide lumen and still overconstricted apices (**C** shows the surface views and **C'** a projection of the surface and invaginated portion). **D,D'** In a matching control placode the invaginated tube (**D'**) displays a narrow lumen and apical surface elongated along the length of the tube (green arrows).

Dotted lines mark the boundary of the placode and the invagination pit is marked by asterisks.

Development • Supplementary information

**E-I** The leading edge during dorsal closure in *apc2*$^{N175K}$ mutant embryos is aberrant, with failed actin accumulation in some areas from stage 14-15, and with some overconstricted and some too relaxed cells at the leading edge itself (**E-G''**; green arrows point to low cortical actin, white arrows to overconstricted cells and magenta arrows to overstretched cells). **H** and **I** show wild-type leading edges at stage 14 and 15, respectively. In all colour panels E-Cadherin is shown in magenta and phalloidin to label F-actin is shown in green, and staining is indicated on single channel panels. All scale bars are 10µm.

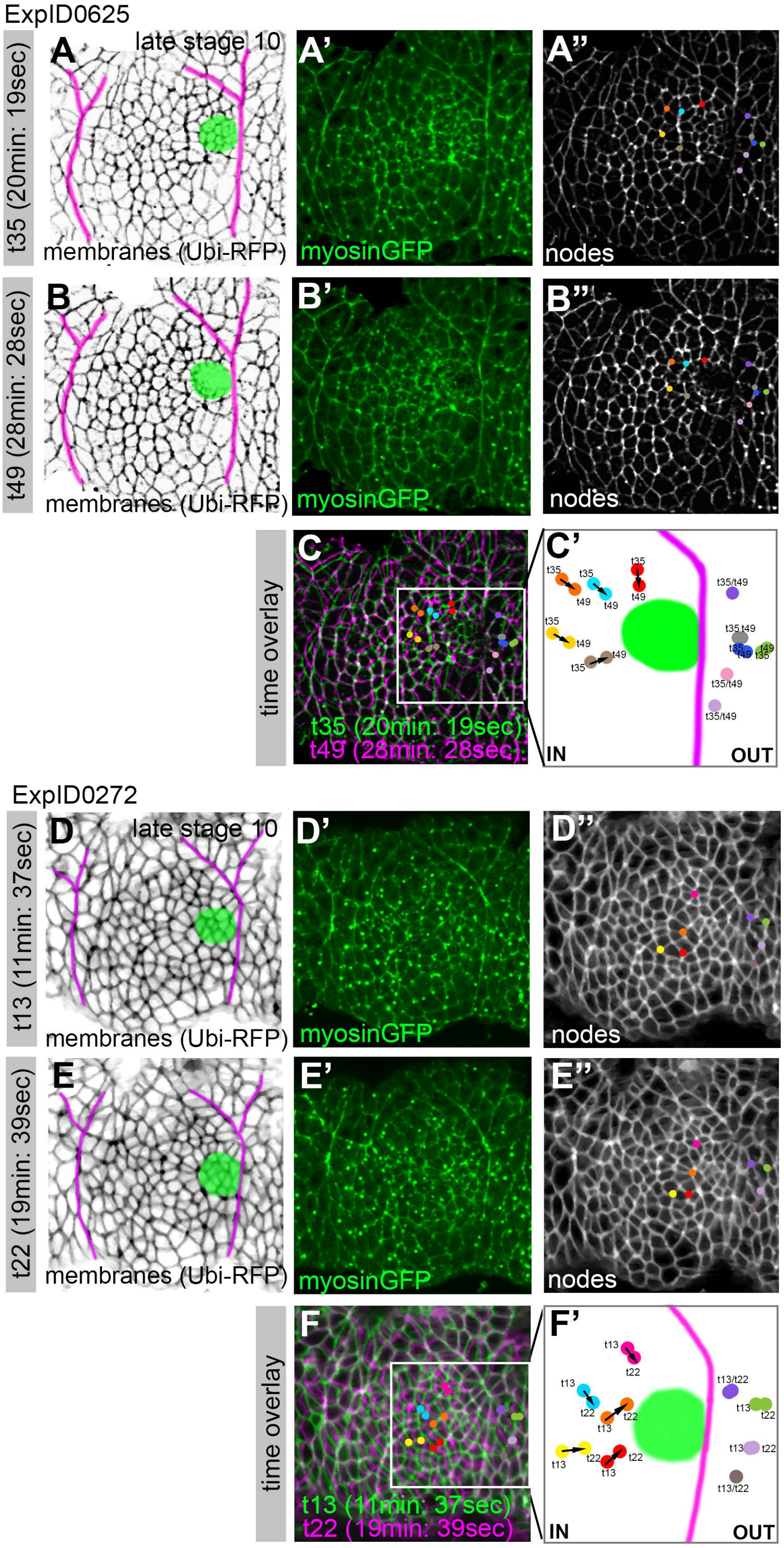


Supplemental Figure S6_Ashour et al.

Development • Supplementary information

**Fig. S6, related to Figure 7. In vivo analysis of the effect of a contractility barrier on cell vertex movement.**

Two further examples of qualitative analyses of cell vertex movement at the salivary gland placode at late stage 10 of vertices near the forming invagination pit where apices constrict, located inside or outside the actomyosin cable. Time-lapse movies were collected of embryos expressing a ubiquitous membrane-tethered RFP (*Ubi-RFP*) as well as a GFP-myosin regulatory light chain (*sqhGFP*) transgene. **A, B** and **D, E** Stills from time-lapse movies (ExpID0625 and ExpID0272), 8min:9sec apart (t35 and t49), and 8min:2secapart (t13 and t22), respectively. The placodal boundary actomyosin cable is marked in magenta and the initial group of constricting cells at late stage 10 marked in green. **A',B'** and **C', D'** Stills of the same movies showing SqhGFP to show myosin. **A'',B''** and **C'', D**'' Vertices inside and outside the actomyosin cable near the constricting zone are marked by coloured dots at both time points. **C, F** show both time points false-coloured and superimposed (first time point in green and second time point in magenta, with individual vertices highlighted for both timepoints). **C', F'** Close-up of the positions of vertices inside and outside the cable at both time points. The cable is marked in magenta and the constricting cells in green.

Development • Supplementary information

**Table S1, related to Figure 3. Proteins identified by mass-spectrometric analysis upon anti-GFP co-immunoprecipitation from Zasp52-GFP-Z embryos in comparison to wt embryos.**

All hits (interactors) identified by the mass-spectrometric analysis, listing Difference and -log(P value), as well as protein IDs and actual gene names as used in *Drosophila melanogaster*. All hits are also manually classified as being involved in cell adhesion, microtubule cytoskeleton, actin cytoskeleton, translation, transcription, muscle development and function or mitochondrial function.

Click here to download Table S1

**Table S2, related to Figure 3. Proteins identified by mass-spectrometric analysis upon anti-GFP co-immunoprecipitation from Armadillo-YFP embryos in comparison to wt embryos.**

All hits (interactors) identified by the mass-spectrometric analysis, listing Difference and -log(P value), as well as protein IDs and actual gene names as used in *Drosophila melanogaster*. All hits are also manually classified as being involved in cell adhesion, microtubule cytoskeleton, actin cytoskeleton, translation, transcription, muscle development and function or mitochondrial function.

Click here to download Table S2

## Table S3. Key resources.

| REAGENT or RESOURCE | SOURCE | IDENTIFIER |
|---|---|---|
| **Antibodies** | | |
| Anti-Apc2 (rabbit), 1:200 | http://dx.doi.org/10.1038/11064 | N/A |
| Anti-Crumbs (mouse), 1:10 | Developmental Studies Hybridoma Bank at the University of Iowa (DSHB) | DSHB Cat#Cq4; RRID: AB_528181 |
| Anti-E-Cadherin (rat), 1:10 | Developmental Studies Hybridoma Bank at the University of Iowa (DSHB) | DSHB Cat#5D3; RRID: AB_528116 |
| Anti-FLAG (mouse), 1:1000 | Sigma | Cat#M2 |
| Anti-GFP (rabbit), 1:500 | Abcam | Cat#ab290 |
| Anti-Patj (guinea pig), 1:300 | http://dx.doi.org/10.1083/jcb.201206064 | N/A |
| Anti-Sidekick (guinea pig), 1:300 | http://dx.doi.org/10.1242/dev.158246 | N/A |
| Anti-Zasp52-N (rabbit), 1:200 | http://dx.doi.org/10.1083/jcb.200707045 | N/A |
| Donkey anti-Goat IgG (H+L) Cross-Adsorbed Secondary Antibody, Alexa Fluor™ 488 | Invitrogen™ | Cat#A-11055 |
| Goat anti-Rabbit IgG (H+L) Cross-Adsorbed Secondary Antibody, Alexa Fluor™ 488 | Invitrogen™ | Cat#A-11008 |
| Goat anti-Guinea Pig IgG (H+L) Highly Cross-Adsorbed Secondary Antibody, Alexa Fluor™ 647 | Invitrogen™ | Cat#A-21450 |
| Goat anti-Mouse IgG (H+L) Cross-Adsorbed Secondary Antibody, Alexa Fluor™ 488 | Invitrogen™ | Cat#A-11001 |
| Cy™5 AffiniPure Donkey Anti-Mouse IgG (H+L) | Jackson ImmunoResearch Laboratories | Cat#715-175-151 |
| Alexa Fluor® 647 AffiniPure Donkey Anti-Rat IgG (H+L) | Jackson ImmunoResearch Laboratories | Cat#712-605-153 |
| **Bacterial and virus strains** | | |
| Library Efficiency™ DH5α Competent Cells | Invitrogen™ | Cat#18263012 |
| BL21(DE3) Competent Cells | Thermo Scientific™ | Cat#EC0114 |
| **Chemicals, peptides, and recombinant proteins** | | |
| GST | Joseph Watson | N/A |
| Rabbit muscle actin | Cytoskeleton | Cat#Akl99 |
| Rhodamine-phalloidin | Cytoskeleton | Cat#PHDR1 |
| Vectashield | Vectorlabs | Cat#H-1000 |
| **Experimental models: Cell lines** | | |
| S2 cells | S2-DRSC (DGRC Stock 181 ; https://dgrc.bio.indiana.edu//stock/181) | RRID:CVCL_Z992 |
| **Experimental models: Organisms/strains** | | |
| *Drosophila melanogaster: apc2$^{D40}$* | Drosophila Bloomington Stock Centre | Cat#6801 |
| *Drosophila melanogaster: apc2$^{N175K}$* | Drosophila Bloomington Stock Centre | Cat#7210 |
| *Drosophila melanogaster: actnCC01961* | Drosophila Bloomington Stock Centre | Cat#51573 |
| *Drosophila melanogaster: enGal4* | Drosophila Bloomington Stock Centre | Cat#1973 |
| *Drosophila melanogaster: Df(2R)BSC308* | Drosophila Bloomington Stock Centre | Cat#23691 |
| *Drosophila melanogaster: hsFLP; ovoD1 /TTP* | Gift from Isabel Palacios | N/A |
| *Drosophila melanogaster: shgTdTomato* | Drosophila Bloomington Stock Centre | Cat#58789 |
| *Drosophila melanogaster: sdk$^{MB05054}$* | Drosophila Bloomington Stock Centre | Cat#24603 |

| | | |
|---|---|---|
| *Drosophila melanogaster: sqh-TagRFPt[3B]* | https://dx.doi.org/10.1016%2Fj.devcel.2019.05.027 | N/A |
| *Drosophila melanogaster: sqh[AX3];::sqhGFP42, UbiRFP-CAAX* | Kyoto Drosophila Genomic Research Centre<br>PMID: 30015616 | Cat#109822 |
| *Drosophila melanogaster: zasp52$^{\Delta}$* | Drosophila Bloomington Stock Centre | Cat#59018 |
| *Drosophila melanogaster: zasp52-GFP[G00189]* | Drosophila Bloomington Stock Centre | Cat#6838 |
| *Drosophila melanogaster: zasp52-GFP[ZCL423]* | Drosophila Bloomington Stock Centre | Cat#58790 |
| *Drosophila melanogaster: armadillo-YFP[CPTI001198]* | Kyoto Stock Centre (DGGR) | Cat#115-134 |
| *Drosophila melanogaster: UAS-Zasp52-PK-6xHis-Flag* | Kind gift of Frieder Schöck; (Liao *et al.*, 2020) | N/A |
| *Drosophila melanogaster: UAS-Zasp52-PR∆PDZ-6xHis-Flag* | Kind gift of Frieder Schöck; (Liao *et al.*, 2020) | N/A |
| **Oligonucleotides** | | |
| Zasp52-PF actin binding site (FseI and AscI sites introduced) residues 1312 – 1319)<br>(see details below due to length) | Synthesised by Twist Bioscience | N/A |
| Zasp52-PF (FseI and AscI sites introduced) residues 1 – 2198)<br>(see details below due to length) | Synthesised by Twist Bioscience | N/A |
| Zasp52-PF PDZ + ZM (FseI and AscI sites introduced) residues 1 – 216)<br>(see details below due to length) | Synthesised by Integrated DNA Technologies | N/A |
| | | |
| **Recombinant DNA** | | |
| pGEX dElp4S FA D338 | kind gift of Emmanuel Derivery; https://doi.org/10.1038/nature16443 | N/A |
| pMT puro FA | kind gift of Emmanuel Derivery; https://doi.org/10.1038/nature16443 | N/A |
| | | |
| | | |
| | | |
| **Software and algorithms** | | |
| Clustal Omega | https://doi.org/10.1093/nar/gkac240 | N/A |
| ImageJ/Fiji | NIH | N/A |
| Imaris | Bitplane | N/A |
| Perseus (MaxQuant) | https://doi.org/10.1038/nmeth.3901 | N/A |
| Prism | Graphpad | N/A |
| Vertex Based Model of Epithelial Mechanics (in house) | DOI: 10.5281/zenodo.7016948<br>(this publication) | N/A |

## Table S4. Embryo genotypes as presented in figures.

| Genotype | Figure | Chromosome | Experiment type |
|---|---|---|---|
| *zasp52-GFP[ZCL423]* | 1B-H’ | 2 | Fixed samples immunofluorescence |
| *zasp52*$^{\Delta}$*/CyO twi::GFP (either mutant or control)* | 2A-B’’ | 2 | Fixed samples immunofluorescence |
| *zasp52-GFP[ZCL423] and zasp52-GFP[G00189]* | 3A | 2 | Co-immunoprecipitations |
| *armadillo-YFP[CPTI001198]* | 3B | 1 | Co-immunoprecipitations |
| *zasp52-GFP[ZCL423]* | 3D-I’ | 2 | Fixed samples immunofluorescence |
| *zasp52*$^{\Delta}$*/CyO twi::GFP (either mutant or control)* | 4A-F | 2 | Fixed samples immunofluorescence |
| *yw hsFLP; FRT G13 zasp52Δ/FRT G13 ovoD x* <br> *zasp52*$^{\Delta}$*/CyO twi::GFP (either mutant or paternally rescued)* | 5A-K | 1,2 | Fixed samples immunofluorescence |
| *zasp52*$^{\Delta}$*/CyO twi::GFP; apc2*$^{D40}$*/TM3 Sb twi::GFP (either mutant or control)* | 6A-K | 2,3 | Fixed samples immunofluorescence |
| *sqh*$^{AX3}$*;sqhGFP; UbiRFP* | 7A-D | 1,2,3 | Live imaging |
| *yw (wild type)* | S1A-E’ | | Fixed samples |
| *zasp52-GFP[ZCL423]* | S2A-B’ | 2 | Fixed samples |
| *enGal4 x UAS-Zasp52-PK-6xHis-Flag* | S3A,A’ | 2,3 | Fixed samples |
| *enGal4 x UAS-Zasp52-PRΔPDZ-6xHis-Flag* | S3B,B’ | 2,3 | Fixed samples |
| *apc2*$^{D40}$*/TM3 Sb twi::GFP (either mutant or control)* | S4A-C’ | 3 | Fixed samples |
| *apc2*$^{N175K}$*/TM3 Sb twi::GFP (either mutant or control)* | S4D-F’’ | 3 | Fixed samples |
| *sqh*$^{AX3}$*;sqhGFP; UbiRFP* | S6 | 1,2,3 | Live imaging |

## Table S5. Binding sites.

| **Zasp52-PF actin binding site (FseI and AscI sites introduced, residues 1312 – 1319)** |
|---|
| ggtgggccggccagagcctcgattgtgtctgccttgaaggaggaaaccgatctggagtaccagaagtatctcaaggcccagcagcgcaacc<br>agaaaagattggactacttccaccagaaagaggaggagctctcgggtctgcagggccaacagctaacccaacttcagagggagctctcga<br>accagcaacagaatcttctgagccaacagcaactgcagcaatccaagttgttgcaactgcagcagtgcgtccagagccaagagttgcagca<br>acaggtgcagcatctcacccagaaatcacaacagcaacctcctcaagctaaccaacagcagcaacaacagcaacagcaacggggtacc<br>caacagcagcaacactcccaagtaacccaaagaacccaacagcaacagcaacaagtgccccaacaagtaacccaacagcaacaaca<br>agaacactctctgctatcgcaaaccacactcgctgagacccaaacccttcaggccaatgcccagtctcagagttccgcttcctacagctccaa<br>agcgactgcttgctctaactcctcttccacagtcggcgcgccggtg |
| |
| **Zasp52-PF (FseI and AscI sites introduced, residues 1 – 2198)** |
| atgcggccggcccatggcccaaccacagctgctgcaaatcaaattgtcacgtttcgatgcccaaccctggggattccgccttcaggggggca<br>cggacttcgctcagcccctgctggtgcaaaaggtgaacgccggcagcttgtccgagcaggctggcctccagcccggcgatgcggtggtcaa<br>gatcaatgacgtggatgtcttcaatctgcgtcacaaggatgcccaggacattgtggtgcgctccggcaacaactttgtcatcacagtgcagcgc<br>ggtggctccacctggcgcccgcatgtgacaccgactggcaatgtgccgcagcccaactcgccgtatctgcagacggtgacgaagacctctct<br>ggctcacaaacaacaggacagccagcacatcggctgtggctacaacaacgcggcccgtcccttctccaacggcggcgatggcggcgtga<br>agagcattgtcaataaacaatacaacaccccggttggcatttacagcgatgaatctattgcggaaacactctcggcccaggcggaggttttgg<br>ctggcggtgtgctcggcgtcaacttcaagaagaacgagaaggaataccagggcgatcgctccgaggttctgaagttcctgcgcgaggagga<br>gaccggccagtccactccagcattcggcaatagccactacgagcatgatgcaccacagcaactgcaacagccacaacagcaatacaacc<br>aacaccagcaacactatcaccagcaacaacaacaacagcaatcgagcaccactcgccatgtcagcgcccccgtgaactcccccaagccc<br>ccgagcaccggcggactcccaactggccagaacatttgcaccgaatgcgagcgcctcattactggcgttttcgtgcgcatcaaggataagaa<br>cctgcacgtggagtgcttcaagtgtgccacgtgtggcacctcgctgaagaaccagggctactacaacttcaacaacaagctctactgcgacat<br>ccacgccaaacaggccgccatcaacaatccccccaccggcaccgagggctacgtccccgttcccatcaagcccaacaccaagctgagtg<br>cctccaccatctcatcggccttgaactcgcacggatacggtggccactcgaacggctactccaatggaaactccacccctgctccggcaccg<br>gttgcaagctctcaagcaacagcaacagtagcaacggtagcaccatccgctgcaacagcagcaactgcagcagcaacaccccaagcag<br>caactgcaacagatagcccagctgcaacagcatcatcatcagacaatatgtcggcctacgtggcagatgagccctcttcgatttatggccaaa<br>ttagcgctgaatcggtggcattggccccaccaccaccacagccacccactgccggcgggggcgatcagccctttgagtacgtcacgctcacc<br>ggcaacgtcatccgcagcgtgcaggctcccggaaagggggcgtgccccagctacaaggtgaaccagggctatgctcgtccgttcggtgccg<br>ccgctcccaagtcgccggtgtcgtatccgccgcagcagcaacagcagtcgccgcgtcccgctcccggtggccaaaacccgtacgccaccct<br>gccccgcagcaatgtgggccaacaaggtggagaggctgtggaggaactgcagccggaattcgaggaggaggattgctatgagatggaca<br>tcgaggtggccctggccgcaagtcgccaatcgcagcgtggctctagtttcacttggccaccgccgcaggatgatagccacttggcacccacc<br>gcggcgcctctttatatccctccaccggagacgcaacatgtggtggtttcgaatccggtgcagcaagtgcctccattgccacctggaggagcaa<br>ctgctcgactagatccgcaacctgtagttggaacctcggccaacggagctccgcagtggcagagttactccgcaccacaactaacaactgcg<br>agtgcccgccaattggcggaacaggagtctagctcggatagctatacatccacctcgaccaccacgaccactacttcggaggagtatcagcg<br>aatgtacgcagcccaggtgcaggcctatcaaatgcaggagcaatctggctcagagttcgattatcaggtggattacgccagtacccaggattc<br>tgtacaggactatccgtccggcaggagaagtgcccaagagtgcgtggactccctagctgtgcccctaagcacctacaagctggtcgatatggt<br>aagggaggttacacccagtcccgtgaccactccaactcaaactcctgctcctgctgctcctacgacccgtcgcgttgtgttcaacgatgagcctg<br>agattaaggagttaccccaactacccgcggaactagagaccataccggaagcctccgaagctgtagaagatcgcgaaggtcttgtaatcga<br>acagcgatgtcagattctggagagtgaacgcaagttccagcccacacccgagatcaagattgagattgccccagtgcgccaaatacctccg |

Development • Supplementary information

accaagattcccaacccaatgcccaaggagtggattaatcccatgattcgagtcttgaccacagccccggaagttcccttccatctggtggagt
gtccttttccccggccctgtggtgatgattttgaagcggaggcagccgccgccgaagcggccaaaactcaagaggttccggaacctcttcctcc
acaagtttctgctgctccaccggcaacagtttccgttgagccatcacctgctcccttgcgggaatctcctccccgcggatcccgactcagccaag
ccatggttactgctcccgagttcgagctcaagttcgcccctcccgctgaccagggcatcccacttccagaggagaccgagccctatatgccac
cacccattgacacgaaaccctatttgagggaggattaccgacccaaatcaccatttgtgagtgctctaaccaccgctcccgatcgtccctttgaa
ggtcactttgataaagatgtgcccatccacatgattgacctgcccacccccaaagagcacctgagcatgtgtgatgccctttgcaccgccccag
aacgtggttacactcccctgaatcccgagaatgctatgcatcgcgtagacgaggagcaaaagcaacaggaactcaagaagcgtgaatttca
ggtgctggatcacgaggaagagctgggaatccgtccggagcctccacagtctgtcgagtactacgaaacgcggagagatcagccacgga
aatcctccgcctttgcagccatgcaagcattccagccatcccgtgaacctttgtcgtcgaacacggtttcgaatgctggaagtgtggccgatacc
ccaagagcctcgattgtgtctgccttgaaggaggaaaccgatctggagtaccagaagtatctcaaggcccagcagcgcaaccagaaaaga
ttggactacttccaccagaaagaggaggagctctcgggtctgcagggccaacagctaacccaacttcagagggagctctcgaaccagcaa
cagaatcttctgagccaacagcaactgcagcaatccaagttgttgcaactgcagcagtgcgtccagagccaagagttgcagcaacaggtgc
agcatctcacccagaaatcacaacagcaacctcctcaagctaaccaacagcagcaacaacagcaacagcaacggggtacccaacagc
agcaacactcccaagtaacccaaagaacccaacagcaacagcaacaagtgccccaacaagtaacccaacagcaacaacaagaacac
tctctgctatcgcaaaccacactcgctgagacccaaacccttcaggccaatgcccagtctcagagttccgcttcctacagctccaaagcgactg
cttgctctaactcctcttccacagtcccacctgccaacacctctaccgctttcgcacctgctccagctccagcacccaccagcatacctgtccgcc
catcagctatcgctgtacaaagtagctactgcagcagccagttcgatgtccacgaactgatcgaggagaccgccgaggagctcgagcactc
ggaggtcctgttcccgccgccctccccgctgagccacctgaccaaacagggcaaagccgtacagtccggcctccacaaggcggacagcat
ccccaaataccagcgcaactggacggtgctacctacccagagtcccattcgcactccggaaccgcaggagctgcgcgagaacgtaccgct
ggcattcgtggatgctccgaaagcaccagttaccagtgactcttccactgtacatagacccattgcccaggttgctgcgccgacaactgtggttg
ctccttcccgggaacgggagaaggagcggcggccccagctgtcggtgcccattattgttgaggatcgatcgggtccagtaacgatggctttcc
aaccgttagacgaactggtgcgaccggatcaggccctgacgcccaccaggccgtacaccccgtcgctgaccaacaagccggctccaattgt
gcccttctaccagacggaggagaagcttgtcttcgaggagtgctcggctacccatgccaggaactacaacgaattgaacgcctcgccttttcca
gacagaacacgttctccggctccgggaccaccgccaaatcccctgaatgccattcgagcaccgagaatgaaggaaccggaaaccaagtc
gaatattctgtcagtttctggaggtcctcgcttgcagacgggctcaataaccactggacagagttaccagggacaacttttggctcactccgagc
agagttcccagtcggccagtcagagctataaccagcaaccggagagaattacggaacaaagggtgggcaacctgaacatccaacagag
ggagcagtcatctcagctgcagcagcaagctcaatcgcagactcagagtcagacacgcagccaggtgggaaatactcaaatcgaaagac
gtcgcaaggtcaccgaggaattcgaacgtacccagagtgctaaaactattgagatccgaactggctcccagtctgtgagtcaatcaaaggcc
cagtcgcagtccatcagccaggcacagacgcaggctcaatcccagtcccagaatcagtcggacacagaacgtcgctcttcgtacggtaaga
caggattcgtggccagtcaggcaaagcgtctgtcctgcatggaggaagagattagcagtctgaccagccaatcgcaggctattagtgcccgg
gcctctgctctcggagagggctgctttcccaacctgagatcgcccacctttgactcgaagtttccacttaagccggctcccgcggagtctatagtt
ccggggtatgcaactgttccggccgccacaaagatgctaacggcaccaccaccgggtttcctgcagcagcagcagcaacagcagcaaag
gtctgccttctcgggctaccaagccacaacttcatcggtgcagcagagctcttttgcgagcagctcaaaagccacaacctcatcgctctcatcct
catcagcatctgcttcagcatcagcatccgtcgcgagatcgtcgcaaagtctaacccaagcttctgctattactaccaccactaataaccaggc
caccacggcctacaggagcagcaatggcagcattaccaagcctaatctggcctcgcggccatccatcgcttccatcacagctccaggatcag
caagtgctcccgctcctgttccatcggcagctccaaccaaagctactgctccattcaaagctccgattgttccaaaatcggtgatagcgaacgc
cgttaacgccgctgctccgcctgcgcccgctgtctttccgccagacctgagcgatttgaacttgaactctaatgtggataattccccaggtgccgg
aggaaagagcgctggcgcctttggagccacctcggcgcccaagaggggcaggggtatcctgaataaggcagccggacccggagtgcgc
atcccactgtgcaacagctgcaatgtgcagatcagaggaccctttatcacggcattgggccgcatctggtgcccggatcatttcatctgcgtgaa
cggcaactgccgtcgtccgctgcaggacattggattcgttgaggagaagggcgatctgtactgcgagtactgtttcgagaagtacctggcgccc

Development • Supplementary information

| |
|---|
| acttgcagcaagtgcgctggcaagatcaagggtgactgtttgaatgccattggcaaacacttccatccggagtgcttcacctgcggccagtgcg<br>gcaagatctttggcaacaggcccttcttcctggaggatggaaacgcgtactgcgaggccgattggaacgagttgttcaccaccaagtgcttcgc<br>ctgcggcttccccgtggaagctggcgacagatgggtggaggccttgaaccacaactaccatagccaatgcttcaactgcacgttctgcaaac<br>agaacctggagggtcagagcttctacaacaagggcggacgtcccttctgcaagaatcacgcgcgctaagggcgcgccatgc |
| |
| **Zasp52-PF PDZ + ZM (FseI and AscI sites introduced, residues 1 – 216)** |
| atggcccaaccacagctgctgcaaatcaaattgtcacgtttcgatgcccaaccctggggattccgccttcagggggcacggacttcgctcag<br>cccctgctggtgcaaaaggtgaacgccggcagcttgtccgagcaggctggcctccagcccggcgatgcggtggtcaagatcaatgacgtgg<br>atgtcttcaatctgcgtcacaaggatgcccaggacattgtggtgcgctccggcaacaactttgtcatcacagtgcagcgcggtggctccacctg<br>gcgcccgcatgtgacaccaactggcaatgtgccgcagcccaactcgccgtatctgcagacggtgacgaagacctctctggctcacaaacaa<br>caggacagccagcacatcggctgtggctacaacaacgcggcccgtcccttctccaacggcggcgatggcggcgtgaagagcattgtcaata<br>aacaatacaacaccccggttggcatttacagcgatgaatctattgcggaaacactctcggcccaggcggaggttttggctggcggtgtgctcgg<br>cgtcaacttcaagaagaacgagaaggaataccagggcgatcgctccgaggttctgaagttcctgcgcgaggaggagaccggccagtccac<br>tcca |

Development • Supplementary information

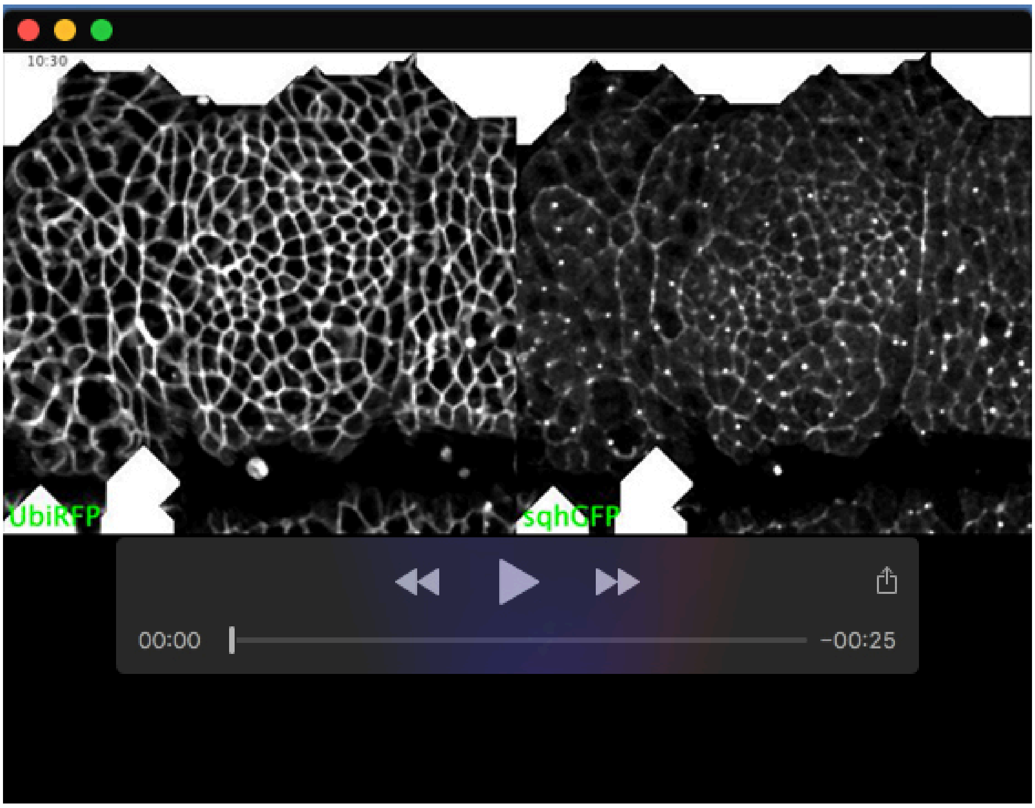


**Movie 1, related to Fig. 7. Analysis of cell vertex movement inside and outside the salivary gland placode boundary.**

Time lapse movie of a *sqhGPF; Ubi-RFP* embryo, ExpID0357, (myosin highlighted by SqhGFP and cell membranes by Ubi-RFP) showing the salivary gland placode and surrounding epidermal cells starting from late stage 10 onwards. Time interval between frames is 1min:30sec. The first and last frame highlight the position of vertices analysed in Figure 7.

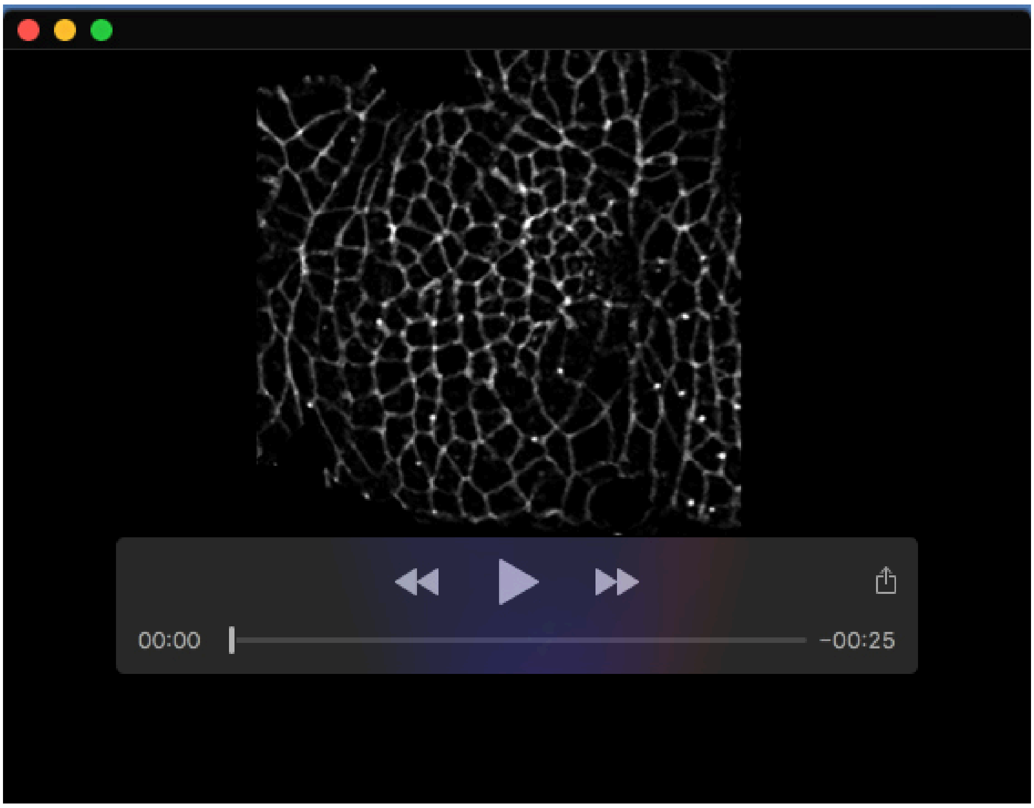


**Movie 2, related to Fig. S6. Analysis of cell vertex movement inside and outside the salivary gland placode boundary.**

Time lapse movie of a *sqhGPF; Ubi-RFP* embryo, ExpID0625, (cell membranes highlighted by Ubi-RFP) showing the salivary gland placode and surrounding epidermal cells starting from late stage 10 onwards. Time interval between frames is 34.8sec. The first and last frame highlight the position of vertices analysed in Supplemental Figure S6.

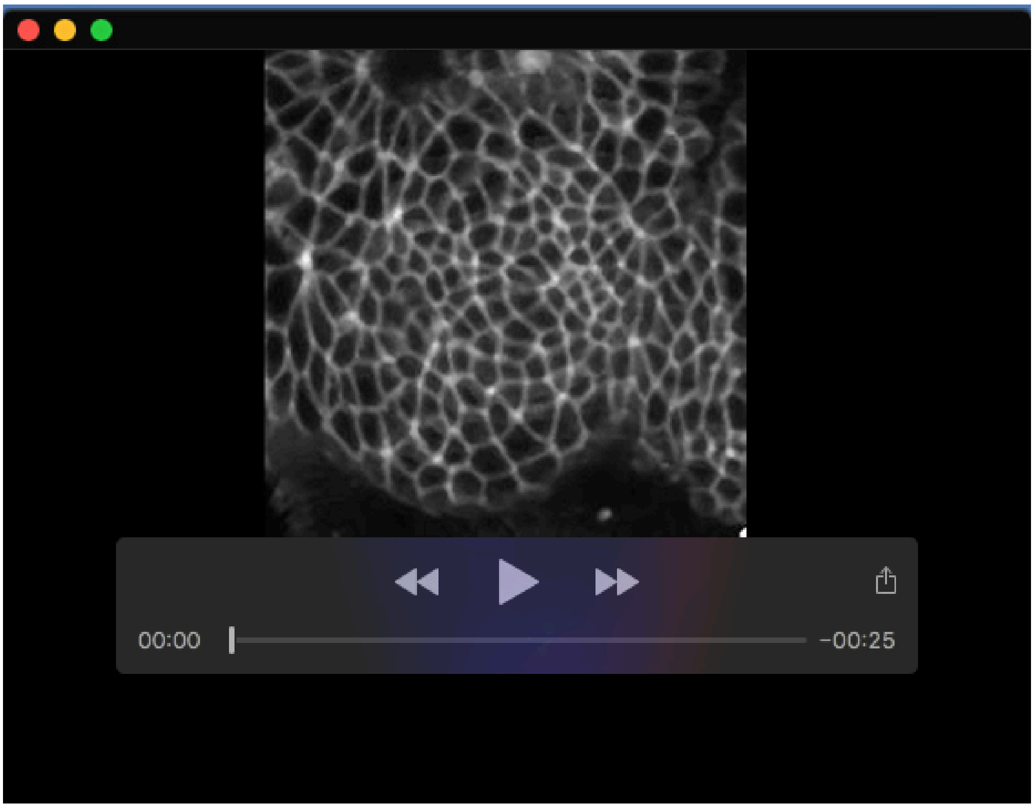


**Movie 3, related to Fig. S6. Analysis of cell vertex movement inside and outside the salivary gland placode boundary.**

Time lapse movie of a *sqhGPF; Ubi-RFP* embryo, ExpID0272, (cell membranes highlighted by Ubi-RFP) showing the salivary gland placode and surrounding epidermal cells starting from late stage 10 onwards. Time interval between frames is 53.6sec. The first and last frame highlight the position of vertices analysed in Supplemental Figure S6.
