# Supplementary material for: Zasp52 strengthens whole embryo tissue integrity through supracellular actomyosin networks
Source: Development. 2023 Apr 3;150(7):dev201238. doi: 10.1242/dev.201238 (PMC10112930; doi:10.1242/dev.201238)
Supplement: Supplementary file 1 [file develop-150-201238_review_history.pdf]

Zasp52 strengthens whole embryo tissue integrity through supracellular actomyosin networks

Dina J. Ashour, Clinton H. Durney, Vicente J. Planelles-Herrero, Tim J. Stevens, James J. Feng and Katja Röper
DOI: 10.1242/dev.201238

Editor: Thomas Lecuit

Review timeline
Original submission: 9 September 2022
Editorial decision: 28 November 2022
First revision received: 20 January 2023
Editorial decision: 21 February 2023
Second revision received: 22 February 2023
Accepted: 28 February 2023

Original submission

First decision letter

MS ID#: DEVELOP/2022/201238

MS TITLE: Zasp52 strengthens whole embryo tissue integrity through supracellular actomyosin networks

AUTHORS: Dina Julia Ashour, Clinton H Durney, Vicente J. Planelles-Herrero, Tim Stevens, James J. Feng, and Katja Röper

I sincerely apologise for the long delay before being to come back to you. I have now received all the referees' reports on the above manuscript, and have reached a decision. The referees' comments are appended below, or you can access them online: please go to BenchPress and click on the 'Manuscripts with Decisions' queue in the Author Area.

As you will see, the referees express considerable interest in your work, but have some criticisms and recommend a revision of your manuscript before we can consider publication. If you are able to revise the manuscript along the lines suggested, which may involve further experiments, I will be happy receive a revised version of the manuscript. Your revised paper will be re-reviewed by one or more of the original referees, and acceptance of your manuscript will depend on your addressing satisfactorily the reviewers' major concerns. Please also note that Development will normally permit only one round of major revision. If it would be helpful, you are welcome to contact us to discuss your revision in greater detail. Please send us a point-by-point response indicating your plans for addressing the referee’s comments, and we will look over this and provide further guidance.

Please attend to all of the reviewers' comments and ensure that you clearly highlight all changes made in the revised manuscript. Please avoid using 'Tracked changes' in Word files as these are lost in PDF conversion. I should be grateful if you would also provide a point-by-point response detailing how you have dealt with the points raised by the reviewers in the 'Response to Reviewers' box. If you do not agree with any of their criticisms or suggestions please explain clearly why this is so.

© 2023. Published by The Company of Biologists under the terms of the Creative Commons Attribution License (https://creativecommons.org/licenses/by/4.0/).

Reviewer 1

Advance summary and potential significance to field

This manuscript by Röper and colleagues identified a role for Zasp52 for supracellular actin cable formation during Drosophila embryogenesis. Supracellular actin cables are a known phenomenon during embryonic patterning that may help coordinating the morphogenesis of many cells in the embryo. They have been prominently observed during gastrulation, dorsal closure or salivary gland placode formation, the latter is the structure the authors focus on here. One suggested role for cable was to mechanically communicate across cell boundaries.
By using an old gene trap in Zasp52 (Zasp52-GFP-ZCL423) that labels most of the more than 20 Zasp protein isoforms, the authors find that Zasp52 is not only present in the sarcomeric Z-disc, a well know place where Zasp52 functions to cross link actin by binding to aActinin, but it is also enriched in supracellular actin cables in the salivary gland placode, the ventral midline at gastrulation or the leading edge during dorsal closure, the latter had already been known from earlier findings.
By homology search the authors find a thus far unidentified actin binding motif (ABM) in the central coil-coil region of Zasp52, included in some of the long Zasp52 isoforms, that is similar to the CapZ-beta actin binding motif, a protein important for barbed (plus) end filament capping. The authors verify that this motif can bind actin in vitro, supposedly stronger than the earlier identified motif at the Zasp N-terminus, although this was not compared in the same experiment.
Biochemical pull-downs suggest that Zasp52 binds to proteins located at epithelial junctions, including Patj Baz, ZO1, Sidekick and APC2, suggesting that Zasp52 might help organizing junctional proteins. This is somewhat supported by the strong Zasp52, APC2 double mutant phenotype.
Zygotic Zasp52 mutants show a rather mild embryonic phenotype, whereas as maternal zygotic Zasp52 loss of function results in severe phenotypes in various tissues. From this, the authors hypothesize that Zasp52 is required for efficient actin cable formation, which in turn will enable the embryo to divide into mechanically different zones that undergo morphogenesis processes that are well insulated from each other. In addition to the experimental data the authors also tried to support this interesting hypothesis by an in silico vertex model that produces morphogenetic domains that are separated by stiff boundaries.
I find this an appealing hypothesis but feel the quantification of phenotypes is rather weak and needs improvement. How many animals were assayed? Which level of phenotypic category was scored and how?
How many tears? How many holes? How many, how long actin cables?

Comments for the author

1. Does the more N-terminal gene trap Zasp52-GFP-G00189 give the same pattern as the ZCL423? The predicted isoforms labelled are not identical for both.

2. The zygotic Zasp52 loss of function phenotype is rather subtle, although the missing supracellular actin cable seems obvious. However, quantification of the ‘cable’ in wild type and Zasp52 would help. Can the cable be tracked?
There are some holes visible in the mutant epidermis in Figure 4A and 4B compared to 4C control. However this is not mentioned. As the phenotypes are subtle, control images are important. However, they are not shown for Figure 4D, so hard to appreciate any difference from wild type. There is also no wild type comparison in Figure S5. It is easy to put some arrows, it would be more useful to support the phenotypes with some numbers.

3. The maternal zygotic phenotype of Zasp52 is very strong, including a partial failure of head involution. To better compare it to maternal null and zygotic plus, as done it Figure 5, the comparable images need to be shown with the same magnifications, here Figure 5B and 5D.
Are the maternal null, zygotic plus embryos wild type? Do they hatch?

4. To better visualize the “large scale disorganization of embryonic tissues” it might be useful to label some with specific markers in the m/z Zasp52 mutants, e.g. the placode if there are early markers, possibly just engrailed stripes might help to better visualize the suggested uncontrolled mix of domains.

© 2023. Published by The Company of Biologists under the terms of the Creative Commons Attribution License (https://creativecommons.org/licenses/by/4.0/).

5. The faster movement of cells inside the pit versus outside the pit is supportive of the suggested function of the actin cable as a mechanical insulator. These movements can easily be quantified. Thus far, Figure 7 shows stills only from a single movie, in which a few cells have been tracked (?). I assume it should be easy to track all cells with tools like tissue analyzer and to this from multiple movies, at least from 3. Is there a difference in the weak Zasp52 zygotic loss of function that supposedly has a strong defect in the actin cable but only a rather weak morphogenesis defect? This would collect more direct support for the hypothesis that the cable insulates mechanically.

6. Modelling: are the predicted weird cell shapes seen in Figure 7F found in vivo? Can the in vivo cell shapes be quantified to support the model with more biological evidence?

Minor
1. Reference formatting: Stronach 2014 bold.
2. Figure 3G is missing the label.
3. Disruption with one ‘s’, Figure 6.
4. It is unclear how movie S1 relates to Figure 7. Can the tracked cells in the figure be labelled in the movie?
What is the membrane label?

<u>Reviewer 2</u>

Advance summary and potential significance to field

This manuscript examines the role of the LIM Domain protein Zasp52 in promoting supracellular actomyosin cable presence. Supracellular actomyosin networks occur as cables along junctions (Martin and Lewis, 1992; Kiehart et al. 2000; Jacinto et al., 2002; Blankenship 2006; Roper 2012) and also as cell surface spanning structures that interconnect cells in more complicated network structure (Martin et al., 2010; Lopez-Gay et al., 2020). A key question about the formation of these supracellular structures is how their connection between cells is regulated. This study first, identified Zasp52 as a component of multicellular actomyosin cables – analyzing two Zasp52::GFP protein trap lines. Zasp52 depletion was show to disrupt F-actin cable formation and the authors identified an F-actin binding domain within the Zasp52 protein that bears similarity to the C-terminus of CapZbeta. The authors test this region by performing an F-actin pelleting assay to show co-precipitation and that Zasp52 interacts with a number of junctional proteins. The authors then showed that Zasp52 mutation disrupts morphogenetic processes at Stage 11 and beyond and interacts genetically with apc2-. Finally, the authors theorize that actomyosin cables are acting a mechanical insulators that ‘protect’ surrounding tissue from morphogenetic processes occurring nearby.

Overall, the characterization of Zasp52’s actin and junctional protein interactions and the finding that it is involved in supracellular actin cable generation will be of importance to the field. The use of Zasp52 mutants to test the role of the actomyosin cable is also of interest. There are some cases where the evidence for conclusions is weak and ways that the authors can strengthen these points.

Comments for the author

Main comments:

1) Fig. 1B-H: Zasp52 localization appears to be on all junctions where it is expressed rather than being specifically localized to multicellular actin cables.
Currently, I would conclude that Zasp52 is junctional and localized to multicellular cables because junctions are also present. Currently, there is no co-staining with myosin II in the main figure (only supplement), this would be better in the main figure to make their point. I think the conclusion would be strengthened by showing enrichment for the multicellular cables. Could the authors score Zasp52 enrichment at multicellular cables by normalizing this intensity to E-cadherin levels at the same junctions? Possibly could compare this score to that of myosin II.

Also, the localization of Zasp52 to TCJs (Fig. 1C’) is not very striking and/or the green circles make it hard to compare junctional/TCJ Zasp52 levels.

© 2023. Published by The Company of Biologists under the terms of the Creative Commons Attribution License (https://creativecommons.org/licenses/by/4.0/).

2) Fig. 2I-J: The Zasp52 colocalization with F-actin in S2 cells is not very convincing. 1) It looks like the Zasp52-ABM-GFP is aggregating significantly when expressed. 2) The colocalization between phalloidin stained F-actin and Zasp52-ABM-GFP is not very striking – i.e. most Zasp52-ABM-GFP is not localized at the cortex and J'' colocalization looks mostly to be a cell filled with Zasp52 aggregates rather than true colocalization. Choosing cells with lower expression and fewer aggregates is important to make this result more convincing.

Fig. 2K-L: The F-actin pelleting assay is sloppy. 1) The amount of G-actin left in the supernatant changes inconsistently from sample to sample, suggesting there is different 'quality' of actin (i.e. amounts of functional actin) between the different pelleting samples. 2) The authors do not show saturation of binding so that one cannot know the Kd of the interaction. Thus, it is unclear how strong this interaction is and whether it would be relevant in vivo.

Fig. 4-7: The authors should quantify the most critical phenotypes that are relevant to their conclusions. For example, the enrichment of actin at the placode boundary should be compared between control and mutant. In addition, some measurement of the shape of the placode between control and mutant would help convince the reader it is different.

Currently, the model is disconnected from the data and analysis. Would be better to measure a property in the data and make a corresponding measure in the model so that they can be directly compared. Comparing the nodal movement of cells outside the placode between wild-type and zasp52 mutant would better provide evidence for there being a insulator effect.

Minor

1) Fig 2K: ABM Ctl is not clear, initially I thought it was ABM pelleting without actin. Should read actin alone or something like that.

2) Fig. 5: Is there a reason the authors switched over to using Crumbs staining rather than E-cadherin?

3) lines 337-342: The result that apc2 mutant enhances the zasp52 mutant does not prove they are working together. If they were working together, then a null mutant in one would have the same effect of mutating both. Presumably the mutants are not completely removing zasp52 and apc function (i.e. maternal contribution) such that they are not true nulls and could synergize if affecting the same pathway. However, the authors can't rule out that these proteins are not functioning in parallel pathways.

First revision

Author response to reviewers' comments

Rebuttal and detailed comments and answers to reviewers' questions

We are pleased that the reviewers find our manuscript of considerable interest, find our hypothesis appealing and think that this study is of importance to the field.
Below, we answer all questions and concerns raised in detail and explain additional experiments and quantification we have undertaken in this revised version of the manuscript.

Reviewer 1 Advance Summary and Potential Significance to Field:
This manuscript by Röper and *colleagues* identified a role for Zasp52 for supracellular actin cable formation during Drosophila embryogenesis. Supracellular actin cables are a known phenomenon during embryonic patterning that may help coordinating the morphogenesis of many cells in the embryo. They have been prominently observed during gastrulation, dorsal closure or salivary gland placode formation, the latter is the structure the authors focus on here. One suggested role for cable was to mechanically communicate across cell boundaries.
By using an old gene trap in Zasp52 (Zasp52-GFP-ZCL423) that labels most of the more than 20 Zasp

© 2023. Published by The Company of Biologists under the terms of the Creative Commons Attribution License (https://creativecommons.org/licenses/by/4.0/).

protein isoforms, the authors find that Zasp52 is not only present in the sarcomeric Z-disc, a well know place where Zasp52 functions to cross link actin by binding to aActinin, but it is also enriched in supracellular actin cables in the salivary gland placode, the ventral midline at gastrulation or the leading edge during dorsal closure, the latter had already been known from earlier findings.
By homology search the authors find a thus far unidentified actin binding motif (ABM) in the central coil-coil region of Zasp52, included in some of the long Zasp52 isoforms, that is similar to the CapZ-beta actin binding motif, a protein important for barbed (plus) end filament capping. The authors verify that this motif can bind actin in vitro, supposedly stronger than the earlier identified motif at the Zasp N-terminus, although this was not compared in the same experiment.
Biochemical pull-downs suggest that Zasp52 binds to proteins located at epithelial junctions, including Patj, Baz, ZO1, Sidekick and APC2, suggesting that Zasp52 might help organizing junctional proteins. This is somewhat supported by the strong Zasp52, APC2 double mutant phenotype.
Zygotic Zasp52 mutants show a rather mild embryonic phenotype, whereas as maternal zygotic Zasp52 loss of function results in severe phenotypes in various tissues. From this, the authors hypothesize that Zasp52 is required for efficient actin cable formation, which in turn will enable the embryo to divide into mechanically different zones that undergo morphogenesis processes that are well insulated from each other. In addition to the experimental data the authors also tried to support this interesting hypothesis by an in silico vertex model that produces morphogenetic domains that are separated by stiff boundaries.
I find this an appealing hypothesis but feel the quantification of phenotypes is rather weak and needs improvement. How many animals were assayed? Which level of phenotypic category was scored and how? How many tears? How many holes? How many, how long actin cables?

Reviewer 1 Comments for the Author:
1. Does the more N-terminal gene trap Zasp52-GFP-G00189 give the same pattern as the ZCL423? The predicted isoforms labelled are not identical for both.

We apologise for not making this clear in the original version of the manuscript. Yes, for the embryonic structures that we analyse and describe both protein trap lines look indistinguishable. We did not state this clearly enough in the original version of the manuscript and have corrected this now.
We attach below a figure for the reviewer to illustrate the labelling using Zasp52[G00189], whereas figures in the paper are showing Zasp52[ZCL423]. We are happy to include this as a further supplemental figure if required, but part of the localization of this line has been published previously (at the amnioserosa/leading edge interface).

This identical localisation is also consistent with the previously published expression analysis by Beth Stronach (B. Stronach / Gene Expression Patterns 15 (2014) 67-79), in which she states: “Finally, immunofluorescence detection of endogenous Zasp52 proteins in embryos with the Zasp52 antisera revealed a pattern of expression and localization indistinguishable from ZCL423 GFP (Fig. 1B)” “Identification of a GFP protein trap line, ZCL423, in the Zasp52 locus, provided a fluorescent tool to visualize endogenous isoforms in many tissues and at different life stages. Comparison of ZCL423 distribution with an extant Zasp52 protein trap (Zasp52G00189) revealed nearly identical expression and localization patterns, with the exception of the ring gland, suggesting that the patterns are largely representative of the endogenous locus.”

© 2023. Published by The Company of Biologists under the terms of the Creative Commons Attribution License (https://creativecommons.org/licenses/by/4.0/).

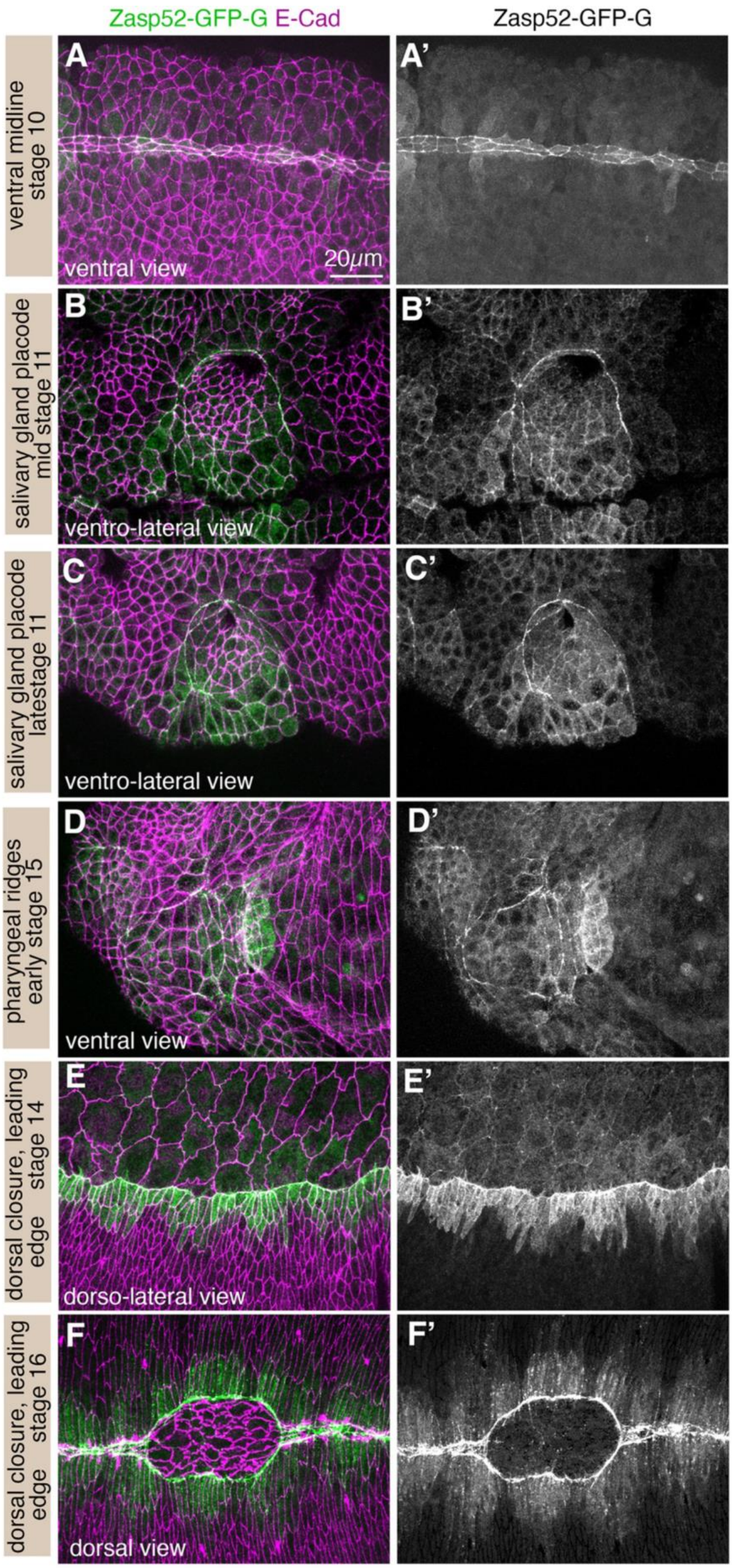

© 2023. Published by The Company of Biologists under the terms of the Creative Commons Attribution License (https://creativecommons.org/licenses/by/4.0/).

2. The zygotic Zasp52 loss of function phenotype is rather subtle, although the missing supracellular actin cable seems obvious. However, quantification of the 'cable' in wild type and Zasp52 would help. Can the cable be tracked?

We quantified the absence of the increased actin in the cable and presented it in Figure 2A-C. We are unsure what additional quantification could be done. The placodal cable is very clearly visible with both phalloidin or when myosin is labelled. We have previously published several manuscripts that also detail the dynamics and behaviour of the cable around the salivary gland placode including its assembly and the molecular mechanism behind the myosin accumulation and activation (Röper, K. Anisotropy of Crumbs and aPKC Drives Myosin Cable Assembly during Tube Formation. Dev Cell ***23****, 939–953 (2012); Sidor, C., Stevens, T. J., Jin, L., Boulanger, J. & Röper, K. Rho-Kinase Planar Polarization at Tissue Boundaries Depends on Phospho-regulation of Membrane Residence Time. Dev Cell* ***52****, 364-378.e7 (2020)). Beyond the above, we are unsure if the reviewer had in mind some other specific kind of tracking.*

There are some holes visible in the mutant epidermis in Figure 4A and 4B compared to 4C control. However, this is not mentioned. As the phenotypes are subtle, control images are important. However, they are not shown for Figure 4D, so hard to appreciate any difference from wild type.

We are sorry that this was not clearly marked in the figure, but what the reviewer identifies as holes are in fact the invagination points of the forming budding tube from the placode (a process detailed and explained in Supplemental Figure S1). We have added more annotation and additional schematic panels to Figure S1 to make the process of cell invagination through the invagination pit clearer. We have also consistently added an asterisks to all panels that show the invagination pit or the position where it is forming to mark it clearly. This was done in S1 but not consistently in all figures, we apologise if this was not clear. The invagination pit is not as clearly visible in panels 4C-C'' as the invagination in the wild-type is forming a more narrow tube and the invagination pit is not as aberrantly wide as in the mutants. We have revised the description of Figure 4 to explain this more clearly.
We have added the matching control for Figure 4D, so the aberrantly wide lumen shape is clearer.

There is also no wild type comparison in Figure S5. It is easy to put some arrows, it would be more useful to support the phenotypes with some numbers.

We have added the corresponding control panels to the figure. All mutant embryos of stage 11 show the overconstricted phenotype and show defects in dorsal closure at stage 14, we now make this clear in the manuscript.

3. The maternal zygotic phenotype of Zasp52 is very strong, including a partial failure of head involution. To better compare it to maternal null and zygotic plus, as done it Figure 5, the comparable images need to be shown with the same magnifications, here Figure 5B and 5D.

We have adjusted the magnifications to be identical.

Are the maternal null, zygotic plus embryos wild type?

The numbers for phenotypes in m-/z- and m-/z+ with regards to the phenotypes we show were listed in the figure legend of the original manuscript including which aspects are rescued in the maternal null paternally rescued embryos.

4. To better visualize the "large scale disorganization of embryonic tissues" it might be useful to label some with specific markers in the m/z Zasp52 mutants, e.g. the placode if there are early markers, possibly just engrailed stripes might help to better visualize the suggested uncontrolled mix of domains.

We do not suggest an uncontrolled mixing of domains. What we argue is that lack or impairment of cable function leads to undue mechanical interference between processes, a reduction in overall structural integrity of epidermal tissues undergoing major morphogenetic changes, and in the case of the salivary gland placode also the loss of the contribution of the circumferential cable to the tube budding process itself.

© 2023. Published by The Company of Biologists under the terms of the Creative Commons Attribution License (https://creativecommons.org/licenses/by/4.0/).

The overall segmented structure of the embryo is unaffected in the m-/z- embryos, as is clearly visible in Fig.5 F, G, H and I.
Only in the head region, and at times the ventral midline region, both areas with prominent actomyosin cables, do we see wide-spread disruption.

5. The faster movement of cells inside the pit versus outside the pit is supportive of the suggested function of the actin cable as a mechanical insulator. These movements can easily be quantified. Thus far, Figure 7 shows stills only from a single movie, in which a few cells have been tracked (?). I assume it should be easy to track all cells with tools like tissue analyzer and to this from multiple movies, at least from 3. Is there a difference in the weak Zasp52 zygotic loss of function that supposedly has a strong defect in the actin cable but only a rather weak morphogenesis defect? This would collect more direct support for the hypothesis that the cable insulates mechanically.

We have now provided analysis of three movies of the relevant time-points, i.e. early apical constriction, comparison of nodes/vertices close to the constricting zone either within the placode or outside it and hence outside the cable. The additional data are now included as Suppl. Fig. S6. All three movies show the same feature: a clear distinction between the movement of nodes inside and outside the actomyosin cable. Those inside the cable, near the forming invagination pit, move towards the pit, whereas those outside the cable at comparable distance move little and in apparently random directions.
Analysis of these movies has to be hand-curated as only early timepoint, prior to proper invagination, can be meaningfully analysed as otherwise the extreme overall tissue curvature at the invagination pit would distort any meaningful analysis. Cells at a greater distance to the pit than the ones analysed also are not meaningful as they undergo further cell behaviours such as directed neighbour exchanges and intercalations, and cells at a greater distance outside the placode are subjected to other morphogenetic events. We have previously segmented, tracked and morphometrically analysed these behaviours in great detail to understand what drive the tube budding process (Sánchez-Corrales, Y. E., Blanchard, G. B. & Röper, K. Radially patterned cell behaviours during tube budding from an epithelium. eLife ***7****, e35717 (2018); Sánchez-Corrales, Y. E., Blanchard, G. B. & Röper, K. Correct regionalization of a tissue primordium is essential for coordinated morphogenesis. eLife* ***10****, e72369 (2021)).*

6. Modelling: are the predicted weird cell shapes seen in Figure 7F found in vivo? Can the in vivo cell shapes be quantified to support the model with more biological evidence?

The in silico model used is a simplified approximation of the placode and the morphogenetic processes that we know are taking place during tube budding (for details see our analyses in (Sánchez-Corrales, Y. E., Blanchard, G.B. & Röper, K. Radially patterned cell behaviours during tube budding from an epithelium. eLife ***7****, e35717 (2018); Sánchez-Corrales, Y. E., Blanchard, G. B. & Röper, K. Correct regionalization of a tissue primordium is essential for coordinated morphogenesis. eLife* ***10****, e72369 (2021); Durney, C. H. & Feng, J. J. A three- dimensional vertex model for Drosophila salivary gland invagination. Phys. Biol.* ***18****, 0-0 (2021)). The model represents the apical constriction of cells that initiates the morphogenesis in vivo, but does not model other behaviours occurring such as cell intercalations and also the invagination, and hence disappearance from the surface, of cells that have reached the pit. Also for simplicity, this in silico model as well as the 3D-vertex model that the one used here is based on (Durney, C. H. & Feng, J. J. A three-dimensional vertex model for Drosophila salivary gland invagination. Phys. Biol.* ***18****, 0-0 (2021)) uses a symmetrical version of the placode with a central pit, whereas in vivo in the wild-type the position of the pit is eccentric (see papers cited above). Therefore, the 'weird cell shapes' are an effect of these simplifications and are not observed as such in vivo.*

Minor
1. Reference formatting: Stronach 2014 bold.
2. Figure 3G is missing the label.
3. Disruption with one 's', Figure 6.

Thanks for spotting these, they have been corrected.

4. It is unclear how movie S1 relates to Figure 7. Can the tracked cells in the figure be labelled in the movie? What is the membrane label?

© 2023. Published by The Company of Biologists under the terms of the Creative Commons Attribution License (https://creativecommons.org/licenses/by/4.0/).

We have now cropped the movie to start with timepoint 3 and end with timepoint 10, the two used in Figure 7. Furthermore we have added the coloured node-label dots to an extra frame of t3 and t10 at the beginning and the end of the movie respectively.

Reviewer 2 Advance Summary and Potential Significance to Field:
This manuscript examines the role of the LIM Domain protein Zasp52 in promoting supracellular actomyosin cable presence. Supracellular actomyosin networks occur as cables along junctions (Martin and Lewis, 1992; Kiehart et al. 2000; Jacinto et al., 2002; Blankenship 2006; Roper 2012) and also as cell surface spanning structures that interconnect cells in more complicated network structure (Martin *et al., 2010; Lopez-Gay et al., 2020). A key question about the formation of these supracellular structures is how their connection between cells is regulated. This study first, identified Zasp52 as a component of multicellular actomyosin cables – analyzing two Zasp52::GFP protein trap lines. Zasp52 depletion was show to disrupt F-actin cable formation and the authors identified an F-actin binding domain within the Zasp52 protein that bears similarity to the C-terminus of CapZbeta. The authors test this region by performing an F-actin pelleting assay to show co- precipitation and that Zasp52 interacts with a number of junctional proteins. The authors then showed that Zasp52 mutation disrupts morphogenetic processes at Stage 11 and beyond and interacts genetically with apc2-. Finally, the authors theorize that actomyosin cables are acting a mechanical insulators that 'protect' surrounding tissue from morphogenetic processes occurring nearby.*

Overall, the characterization of Zasp52's actin and junctional protein interactions and the finding that it is involved in supracellular actin cable generation will be of importance to the field. The use of Zasp52 mutants to test the role of the actomyosin cable is also of interest. There are some cases where the evidence for conclusions is weak and ways that the authors can strengthen these points.

Reviewer 2 Comments for the Author:
Main comments:

1) Fig. 1B-H: Zasp52 localization appears to be on all junctions where it is expressed rather than being specifically localized to multicellular actin cables. Currently, I would conclude that Zasp52 is junctional and localized to multicellular cables because junctions are also present.

We agree with the reviewer's assessment, that -as we discuss in the manuscript- Zasp52 has a propensity to localise to junctions, but that in the locations where supracellular actomyosin cables assemble (downstream of molecular mechanisms that are only in some cases understood) Zasp52 accumulation increases drastically over time. This is very clear for the cable at the leading edge/amnioserosa interface and also for the cable surrounding the salivary gland placode.

Currently, there is no co-staining with myosin II in the main figure (only supplement), this would be *better in the main figure to make their point. I think the conclusion would be strengthened by showing enrichment for the multicellular cables. Could the authors score Zasp52 enrichment at multicellular cables by normalizing this intensity to E-cadherin levels at the same junctions? Possibly could compare this score to that of myosin II.*

We have now added quantifications for Zasp52 versus E-Cadherin enrichment for both the salivary gland cable at stage 11 as well as cables in the head region near the pharyngeal ridges at stage 14. We have performed these quantification exactly as in our previous studies where we analysed the mechanism leading to salivary gland placode cable assembly, quantifying the anisotropy or unequal distribution of components at the plasma membrane of cells at a boundary that forms a cable. As illustrated now in Figure 1I, we quantify the fluorescence intensity ratio of a junction that is part of the cable versus the same cell's adjacent non-cable or side junction.
*This was performed for the same junctions for both Zasp52-GFP and E-Cadherin. The enrichment of Zasp52 in cable over side junctions of around 2 is in the same range and similar to the enrichment of other components of the salivary gland cable that we previously analysed (myosin, ROK; Röper, K. Anisotropy of Crumbs and aPKC Drives Myosin Cable Assembly during Tube Formation. Dev Cell **23**, 939-953 (2012); Sidor, C., Stevens, T. J., Jin, L., Boulanger, J. & Röper, K. Rho-Kinase Planar Polarization at Tissue Boundaries Depends on Phospho- regulation of Membrane Residence Time. Dev Cell **52**, 364-378.e7 (2020)).*

© 2023. Published by The Company of Biologists under the terms of the Creative Commons Attribution License (https://creativecommons.org/licenses/by/4.0/).

The enrichment of Zasp52GFP in cable structures has also been previously commented on and analysed (Stronach, B. Extensive nonmuscle expression and epithelial apicobasal localization of the Drosophila ALP/Enigma family protein, Zasp52. Gene Expression Patterns ***15****, 67–79 (2014); Ducuing, A. & Vincent, S. The actin cable is dispensable in directing dorsal closure dynamics but neutralizes mechanical stress to prevent scarring in the Drosophila embryo. Nat Cell Biol (2016) doi:10.1038/ncb3421).*

Also, the localization of Zasp52 to TCJs (Fig. 1C') is not very striking and/or the green circles make it hard to compare junctional/TCJ Zasp52 levels.

We have removed the green circles and instead point at tri-cellular junctions with arrows which we hope makes the increased intensity at TCJs clearer to see.

2) Fig. 2I-J: The Zasp52 colocalization with F-actin in S2 cells is not very convincing. 1) It looks like the Zasp52- ABM-GFP is aggregating significantly when expressed.
We agree that the overexpression of either theZasp52-ABM or the Zasp52-PF isoform in S2 cells lead to aggregation, and we now mention this explicitly in the results. In our experience such aggregation happens frequently with proteins overexpressed in S2 cells.

2) The colocalization between phalloidin stained F-actin and Zasp52-ABM-GFP is not very striking – i.e. most Zasp52-ABM-GFP is not localized at the cortex and J'' colocalization looks mostly to be a cell filled with Zasp52 aggregates rather than true colocalization. Choosing cells with lower expression and fewer aggregates is important to make this result more convincing.

We agree that the images are not perfect, but feel they are adequate, and would like to point out that apart from the spot like non-specific aggregates, Zasp52-PF-GFP seems to clearly be enriched at the cortex, clearly visible in the higher magnification.
S2 cells do not have very prominent actin structures apart from cortical actin, as visualised in the phalloidin panels.

Fig. 2K-L: The F-actin pelleting assay is sloppy. 1) The amount of G-actin left *in the supernatant changes inconsistently from sample to sample, suggesting there is different 'quality' of actin (i.e. amounts of functional actin) between the different pelleting samples. 2) The authors do not show saturation of binding so that one cannot know the Kd of the interaction. Thus, it is unclear how strong this interaction is and whether it would be relevant in vivo.*

We completely agree that what we are showing is rather a binary (does bind/does not bind) assay and of course without reaching saturation we cannot conclude any Kd from these experiments and are not trying to. We have therefore removed the panel in L.
We would like to argue that the combination of a strong suggestion of direct F-actin binding in vitro (through two different domains) plus colocalization in S2 cells and even more so in vivo, in combination with the conservation of the protein sequence strongly supports a direct binding of Zasp52 to F-actin.

Fig. 4-7: The authors should quantify the most critical phenotypes that are relevant to their conclusions. For example, the enrichment of actin at the placode boundary should be compared between control and mutant.

We have quantified the F-actin reduction at the boundary for the zygotic zasp52 mutant in Figure 2. For both maternal zygotic zasp52 as well as the apc2 zasp52 double mutant, the general tissue disorganisation makes the determination of cells at e.g. the salivary gland placode boundary less reliable, so we opted instead in the maternal zygotic mutants to count the phenotypes that result from boundary and/or tissue integrity disruption.
These are listed in the figure legend.

In addition, some measurement of the shape of the placode between control and mutant would help convince the reader it is different.

© 2023. Published by The Company of Biologists under the terms of the Creative Commons Attribution License (https://creativecommons.org/licenses/by/4.0/).

*We have in previous analyses carefully analysed which highly patterned behaviours of the placodal cells drive wild-type invagination of the tube (Sánchez-Corrales, Y. E., Blanchard, G. B. & Röper, K. Radially patterned cell behaviours during tube budding from an epithelium. eLife **7**, e35717 (2018); Sánchez-Corrales, Y. E., Blanchard, G. B. & Röper, K. Correct regionalization of a tissue primordium is essential for coordinated morphogenesis. eLife **10**, e72369 (2021)). A clear indication of aberrant cell behaviours in the placode is the invaginated tube itself. We observed aberrant lumens already in early invagination, but also beyond, in both zygotic and maternal/zygotic zasp2 mutants (as shown in cross-sections in Fig. 4B'' inset, D and D'; Fig. 5B'' inset, E' plus inset).*

Currently, the model is disconnected from the data and analysis. Would be better *to measure a property in the data and make a corresponding measure in the model so that they can be directly compared. Comparing the nodal movement of cells outside the placode between wild-type and zasp52 mutant would better provide evidence for there being a insulator effect.*

We are sorry if the connection between model and data was not clear enough.
We have rewritten the section of the results describing Figure 7 to make the link clearer.

*In vivo, now illustrated with data from three movies (see reviewer 1), we can see an indication of insulator function through the analysis of nodal movement inside and outside the placode boundary cable as a result of apical constriction at the forming invagination pit. Furthermore we know from previous studies that the boundary cable is under increased tension (laser ablation experiments; Röper, K. Anisotropy of Crumbs and aPKC Drives Myosin Cable Assembly during Tube Formation. Dev Cell **23**, 939-953 (2012)). We cannot formally exclude, though, that the observed differences could be due to some other differences between placodal cells and surrounding epidermal cells. Hence the model allows us to test if this difference in nodal movement observed in vivo could really be the result of only an increased spring constant that an actomyosin cable would provide compared to a normal junction. Hence, the model tests, in a simplified version of the placode, the generality of the hypothesis. Therefore, we feel there is a strong connection between in vivo observations and use of the model.*

Minor

1) Fig 2K: ABM Ctl is not clear, initially I thought it was ABM pelleting without actin. Should read actin alone or something like that.

Thanks for pointing this out, it has been changed.

2) Fig. 5: Is there a reason the authors switched over to using Crumbs staining rather than E-cadherin?

In our experience, both E-Cadherin and Crumbs labelling can serve equally well in delineating apical junctional borders of epithelial cells in the fly embryo. As the antibodies are raised in different species (rat and mouse, respectively), the use of one versus the other is usually dictated by the combination of antibodies used in our stainings that tend to be triple labelled (even if not all channels are always shown). We have used both antibodies interchangeably in various previous studies.

3) lines 337-342: The result that apc2 mutant enhances the zasp52 mutant does not prove they are working together. If they were working together, then a null mutant in one would have the same effect of mutating both. Presumably the mutants are not completely removing zasp52 and apc function (i.e. maternal contribution) such that they are not true nulls and could synergize if affecting the same pathway. However, the authors can't rule out that these proteins are not functioning in parallel pathways.

We agree. The zasp52 zygotic mutant, as we illustrate here through the mutant phenotype in comparison to the maternal zygotic mutant phenotypes is clearly not a null, and Apc2 also shows maternal expression (acc. to public databases), so is likely also not a null mutant.
The enhancement of the phenotype in the apc2 zasp52 double mutant, as the reviewer suggest, is compatible with both working in the same pathway/complex that each mutant only partially

© 2023. Published by The Company of Biologists under the terms of the Creative Commons Attribution License (https://creativecommons.org/licenses/by/4.0/).

disturbs, but is not proof of a direct working together.
We cannot completely exclude that they work in 'parallel pathways' whose effects add up in the double mutant, but the corroborating facts that the proteins can be co-IPed and that the double mutant embryos very much resemble the maternal zygotic mutant zasp52 embryos suggests to us that they might indeed work together.

We have adjusted the phrasing in the results and discussion to reflect this better.

Second decision letter

MS ID#: DEVELOP/2022/201238

MS TITLE: Zasp52 strengthens whole embryo tissue integrity through supracellular actomyosin networks

AUTHORS: Dina Julia Ashour, Clinton H Durney, Vicente J. Planelles-Herrero, Tim Stevens, James J. Feng, and Katja Röper

I have now received all the referees reports on the above manuscript, and have reached a decision. The referees' comments are appended below, or you can access them online: please go to BenchPress and click on the 'Manuscripts with Decisions' queue in the Author Area.

The overall evaluation is positive and we would like to publish your manuscript in Development. Before we can proceed with this, can you please address the few minor text/figure edits suggested by reviewer 1. No further data analysis is required.

Reviewer 1

Advance summary and potential significance to field

This revised manuscript by Röper and colleagues has been improved. It now shows wild type next to mutant at comparable magnifications that readers can appreciate the different phenotypes. Just referring to other published papers, as done before, does not help the non-expert reader.

I had mainly criticized the very little/absent quantifications of actin cables, Zasp52 localization and mutant phenotypes in the initial manuscript. Thanks for now adding the quantification of the Zasp52 enrichment on the cables in Figure 1 and comparing to E-Cad. I had initially missed the change in cable intensity judged by phalloidin stain, comparing wild type to Zasp52 mutants, thanks for making this clear.

I still think the authors should do some work in quantifying the movies shown in Figure 7 that address the main point of the manuscript, cells outside the placode move less than the ones inside. Comparing numbers to Zasp52 mutants would make the manuscript significantly stronger.

Comments for the author

1. Thanks for now supporting the two different Zasp-GFP traps with images. I suggest to include the second one in the supplement of the manuscript. As both traps label different Zasp52 isoforms, this information is interesting.

2. Thanks for making clear to this reviewer that the quantification of the Zasp52 m/z phenotypes are in the figure legend. Please include them in the figure itself, as other readers might miss them too.
3. The faster movement of cells inside the placode versus outside is supportive of the suggested function of the actin cable as a mechanical insulator. Thanks for now recording three movies

© 2023. Published by The Company of Biologists under the terms of the Creative Commons Attribution License (https://creativecommons.org/licenses/by/4.0/).

instead of one and comparing the cells inside and outside the placode qualitatively in the Supplement. Why can the cell movements not be quantified and the result displayed with numbers? I assume the labelling of Figure 7A and B, t3 vs t10 was mixed.

Please provide the 2 other movies as supplementary data.
Movies of Zasp52 mutants would be a strong asset to the paper.

Reviewer 2

Advance summary and potential significance to field

Same as 1st review.

Comments for the author

I am satisfied with the changes the authors made in response to first review.

Second revision

Author response to reviewers' comments

We have implemented the last suggestions by reviewer 1, i.e. added the phenotype quantifications to the GLC figure, added the two further supplemental movies and added the images of the second protein trap line in Zasp52 (Zasp52-GFP-G) to Supplemental Figure S2.

Third decision letter

MS ID#: DEVELOP/2022/201238

MS TITLE: Zasp52 strengthens whole embryo tissue integrity through supracellular actomyosin networks

AUTHORS: Dina Julia Ashour, Clinton H Durney, Vicente J. Planelles-Herrero, Tim Stevens, James J. Feng, and Katja Röper
ARTICLE TYPE: Research Article

I am happy to tell you that your manuscript has been accepted for publication in Development, pending our standard ethics checks.

© 2023. Published by The Company of Biologists under the terms of the Creative Commons Attribution License (https://creativecommons.org/licenses/by/4.0/).
